# Supplementary figures and images for: Trends and disparities in antenatal care utilization: A multilevel analysis of four-plus and eight-plus ANC visits among urban women in Ethiopia
Source: PLoS One. 2025 Nov 24;20(11):e0337241. doi: 10.1371/journal.pone.0337241 (PMC12643277; doi:10.1371/journal.pone.0337241)

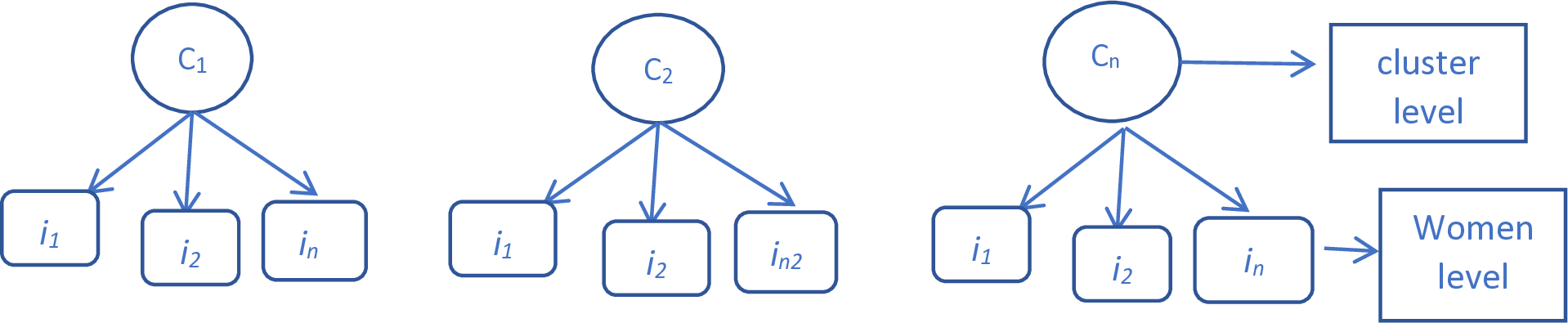

Supplement: S1 Fig — (TIF) [file pone.0337241.s001.tif]

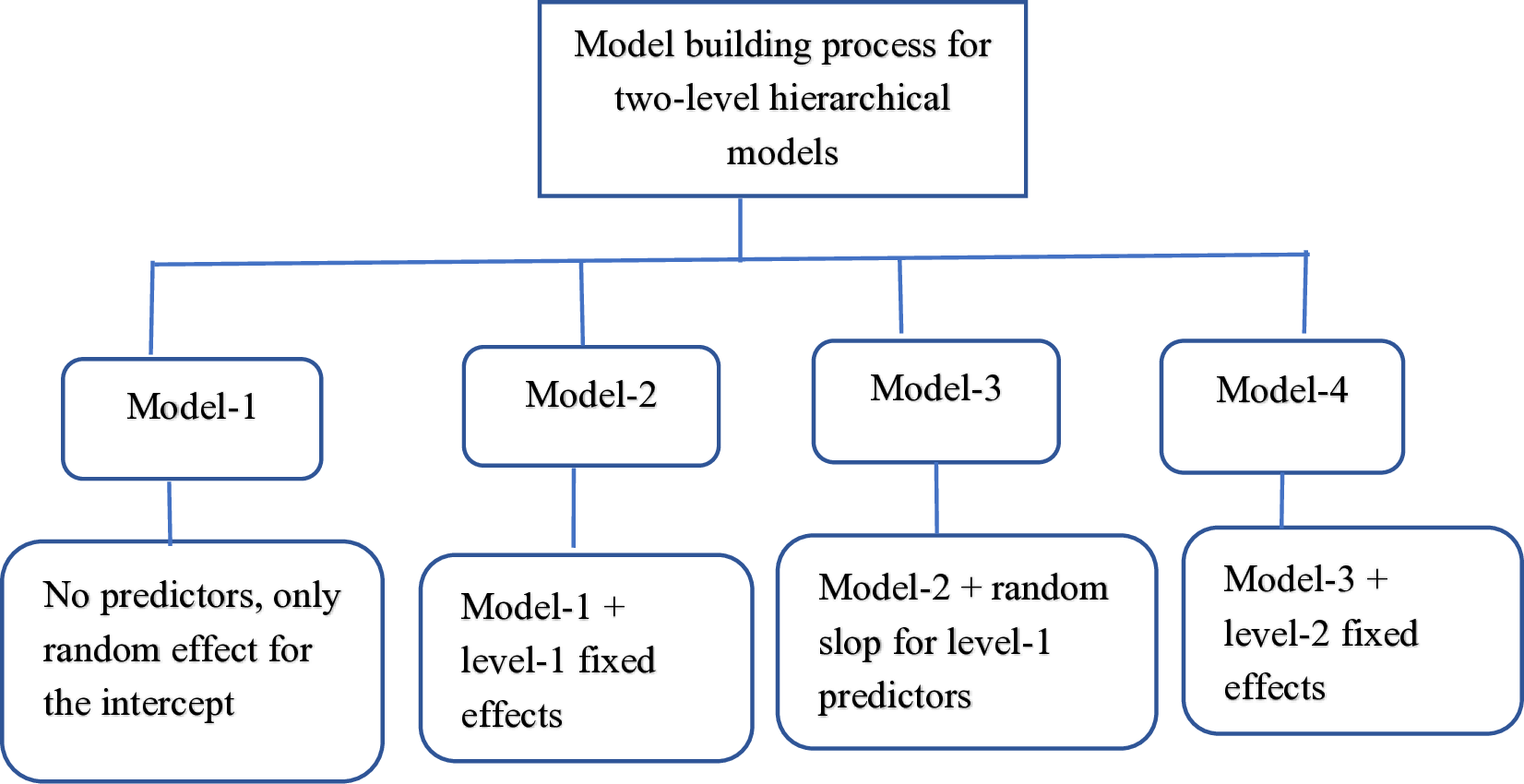

Supplement: S2 Fig — (TIF) [file pone.0337241.s002.tif]

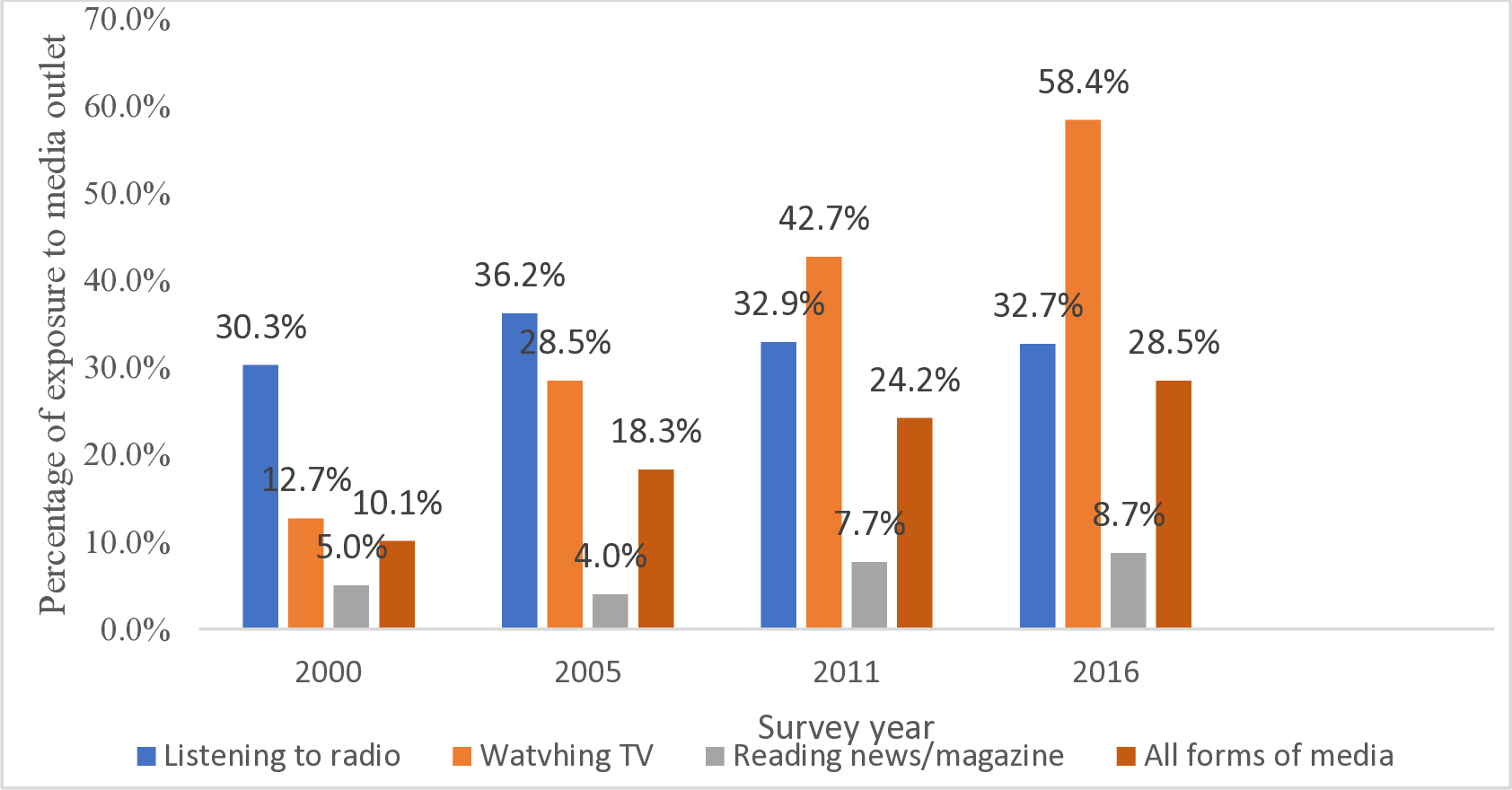

Supplement: S3 Fig — (TIF) [file pone.0337241.s003.tif]

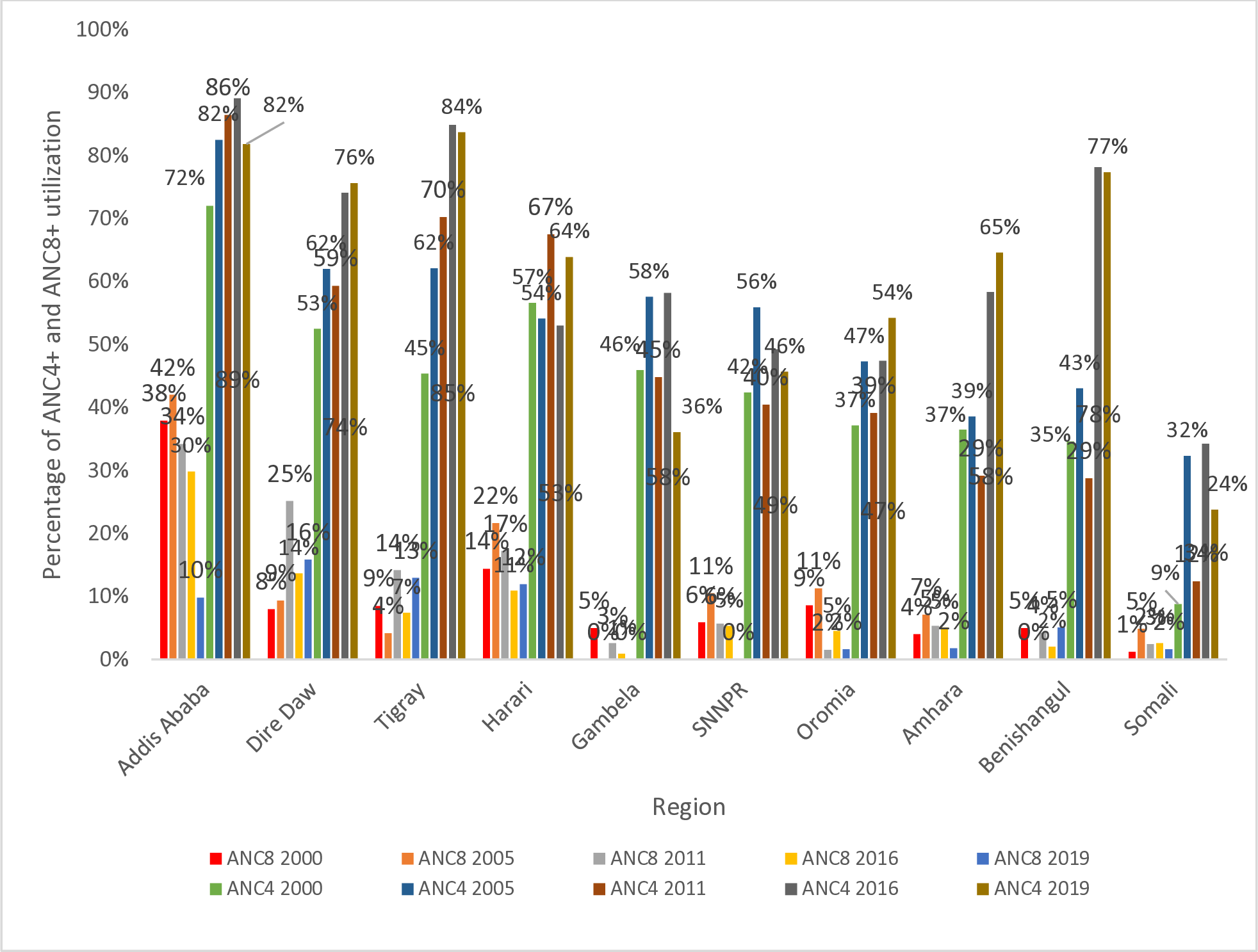

Supplement: S4 Fig — (TIF) [file pone.0337241.s004.tif]

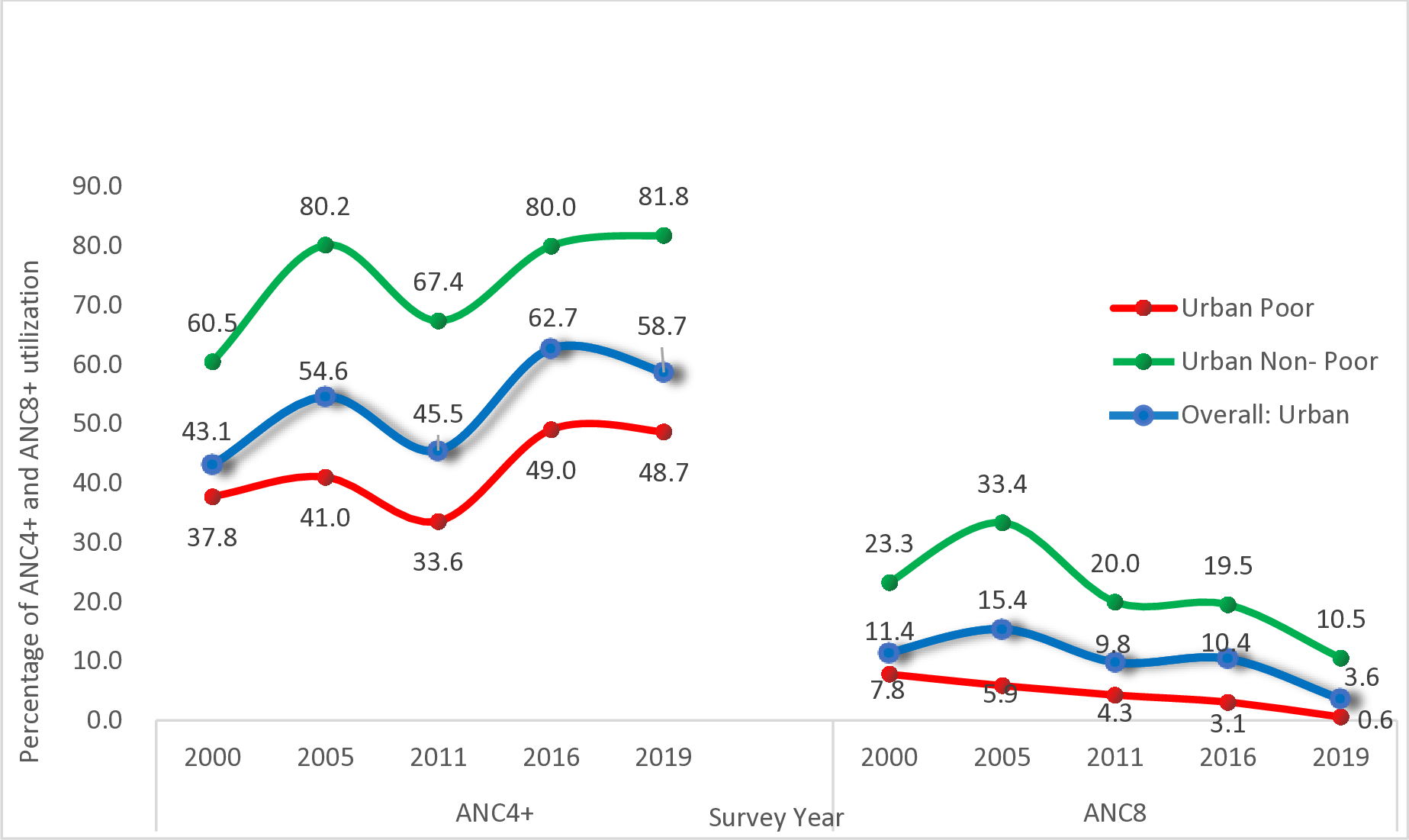

Supplement: S5 Fig — (TIF) [file pone.0337241.s005.tif]

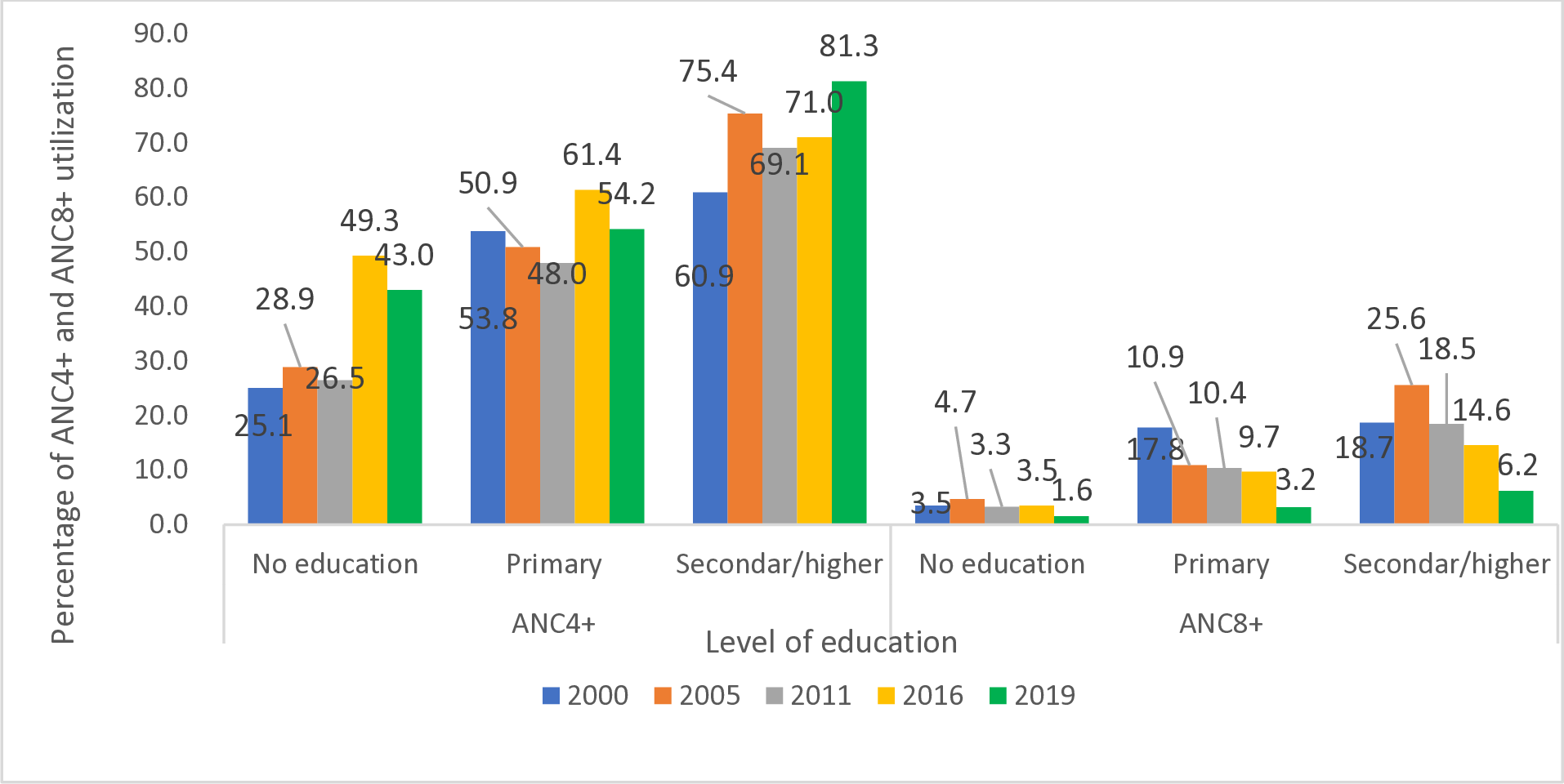

Supplement: S6 Fig — (TIF) [file pone.0337241.s006.tif]
