# Supplementary material for: Trends and disparities in antenatal care utilization: A multilevel analysis of four-plus and eight-plus ANC visits among urban women in Ethiopia
Source: PLoS One. 2025 Nov 24;20(11):e0337241. doi: 10.1371/journal.pone.0337241 (PMC12643277; doi:10.1371/journal.pone.0337241)
Supplement: Attachment — Submitted filename: PONE-D-25-24967.pdf [file pone.0337241.s008.pdf]

PLOS ONE

Trends and Disparities in Antenatal Care Utilization: A Multilevel Analysis of Four and Eight ANC Visits among Urban Women in Ethiopia

--Manuscript Draft--

Manuscript Number:	PONE-D-25-24967
Article Type:	Research Article
Full Title:	Trends and Disparities in Antenatal Care Utilization: A Multilevel Analysis of Four and Eight ANC Visits among Urban Women in Ethiopia
Short Title:	Trends and Disparities in Antenatal Care Utilization
Corresponding Author:	Worku Dechassa Heyi, MPH<br>Addis Ababa University<br>Addis Ababa, ETHIOPIA
Keywords:	Trends, ANC4+, ANC8+, Utilization, Urban, poor, women in RH group, Ethiopia.
Abstract:	Introduction: Antenatal care is the gateway of the continuum of reproductive health care, offering a framework for critical healthcare functions such as complication prevention, screening, diagnosis, and health promotion.<br>Objective: This study examined disparities and trends of four plus and eight plus antenatal care utilization in urban Ethiopia between 2000 and 2019.<br>Methods: Datasets from the 2000, 2005, 2011, 2016, and 2019 Ethiopian Demographic and Health Surveys were analyzed using Stata version 16.1. Analyses were disaggregated by wealth status, education, and region. Multilevel logistic regression analysis was used to identify factors associated with four plus and eight plus antenatal care utilization. Significance was assessed using adjusted odds ratio(AOR) and 95% confidence intervals at p<0.05.<br>Results: Four or more antenatal care(ANC4+) utilization increased from 43.1% in 2000 to 58.7% in 2019(+15.6 points), while, eight or more antenatal care (ANC8+) utilization decreased from 11.4% in 2000 to 3.6% in 2019(-7.8 points). Among urban non-poor, ANC4+ or more increased from 60.5% in 2000 to 81.8% in 2019. While the utilization among urban poor over 20 years changed from 37.8% in 2000 to 48.7% in 2019. Concerning the regional variation, Addis Ababa recorded the highest ANC4+ utilization, increasing from 72% in 2000 to 82% in 2019 a 10 percentage point increase. In contrast, ANC8+ utilization in the region declined sharply, from 38% in 2000 to 10% in 2019, marking a 28 percentage point decrease. The Somali region had the lowest ANC4+ utilization, though it increased from 9% in 2000 to 24% in 2019 a 15 percentage point increase. Meanwhile, Gambella had the lowest ANC8+ utilization, dropping from 5% in 2000 to 0% in 2019.<br>Wealth disparities were also pronounced; urban non-poor women had significantly higher odds of utilizing both ANC4+ (AOR: 2.43, 95% CI: 2.12–2.78) and ANC8+ (AOR: 3.02, 95% CI: 2.49–3.65) compared to urban poor women. Similarly, significant differences were observed by maternal education, with women with secondary or higher education more likely to utilize ANC services (ANC4+: AOR: 3.02, 95% CI: 2.54–3.59; ANC8+: AOR: 2.13, 95% CI: 1.63–2.78). Women aged 30-34 years had higher odds of utilizing ANC4+ (AOR: 2.89, 95% CI: 2.10–3.98) and ANC8+ (AOR: 2.22, 95% CI: 1.37–3.59). Additionally, media exposure at least once a week was positively associated with increased utilization of ANC services, with women having 1.53 times higher odds of utilizing ANC4+ (AOR: 1.53, 95% CI: 1.28–1.83).<br>Conclusion: This study showed the significant regional, wealth, education disparities in ANC utilization in urban Ethiopia. Wealth status was the strongest predictor of ANC utilization, with urban non-poor women more likely to utilize ANC4+ and ANC8+ services. Maternal education, age, media exposure, and marital status also showed positive associations, while regional disparities persisted, with Addis Ababa outperforming other urban areas. To enhance ANC utilization, particularly among urban poor women, targeted intervention, media awareness creation, and educational initiatives should be prioritized.
Order of Authors:	Worku Dechassa Heyi, MPH
	Wubegzier Mekonnen Ayele, PhD

Powered by Editorial Manager® and ProduXion Manager® from Aries Systems Corporation

	Dessalegn Y Melesse, PhD
**Opposed Reviewers:**	Tariku Tesfaye Bekuma, MPH<br>Assistant Professor, Wollega University<br>tarii2007@gmail.com<br>He is an academician and also serve as reviewre for PloS ONE.
	Hailu Fekadu Demissie, PhD<br>Assistant Professor, Arsi University<br>hailufekadu18@yahoo.com<br>He is a PhD holder and an academician
**Additional Information:**	
**Question**	**Response**
**Financial Disclosure**<br><br>Enter a financial disclosure statement that describes the sources of funding for the work included in this submission. Review the submission guidelines for detailed requirements. View published research articles from *PLOS ONE* for specific examples.<br><br>This statement is required for submission and **will appear in the published article** if the submission is accepted. Please make sure it is accurate.<br><br>**Funded studies**<br>Enter a statement with the following details:<br>• Initials of the authors who received each award<br>• Grant numbers awarded to each author<br>• The full name of each funder<br>• URL of each funder website<br>• Did the sponsors or funders play any role in the study design, data collection and analysis, decision to publish, or preparation of the manuscript?<br><br>Did you receive funding for this work?	The author(s) received no specific funding for this work.
**Competing Interests**<br><br>Use the instructions below to enter a competing interest statement for this submission. On behalf of all authors, disclose any competing interests that could be perceived to bias this work—acknowledging all financial support and any other relevant financial or non-	The authors have declared that no competing interests exist.

financial competing interests.<br><br>This statement is required for submission and **will appear in the published article** if the submission is accepted. Please make sure it is accurate and that any funding sources listed in your Funding Information later in the submission form are also declared in your Financial Disclosure statement.<br><br>View published research articles from *PLOS ONE* for specific examples.<br><br>**NO authors have competing interests**<br><br>Enter: *The authors have declared that no competing interests exist.*<br><br>**Authors with competing interests**<br><br>Enter competing interest details beginning with this statement:<br><br>*I have read the journal's policy and the authors of this manuscript have the following competing interests: [insert competing interests here]*<br><br>* typeset	
**Ethics Statement**<br><br>Enter an ethics statement for this submission. This statement is required if the study involved:<br><br>• Human participants<br>• Human specimens or tissue<br>• Vertebrate animals or cephalopods<br>• Vertebrate embryos or tissues<br>• Field research<br><br>Write "N/A" if the submission does not require an ethics statement.<br><br>General guidance is provided below.<br>Consult the submission guidelines for	Written ethical approval for the study was obtained from the institutional review board of the College of Health Sciences of Addis Ababa University with protocol no.040/24/SPH

Powered by Editorial Manager® and ProduXion Manager® from Aries Systems Corporation

detailed instructions. **Make sure that all information entered here is included in the Methods section of the manuscript.**<br><br>**Format for specific study types**<br><br>**Human Subject Research (involving human participants and/or tissue)**<br>• Give the name of the institutional review board or ethics committee that approved the study<br>• Include the approval number and/or a statement indicating approval of this research<br>• Indicate the form of consent obtained (written/oral) or the reason that consent was not obtained (e.g. the data were analyzed anonymously)<br><br>**Animal Research (involving vertebrate animals, embryos or tissues)**<br>• Provide the name of the Institutional Animal Care and Use Committee (IACUC) or other relevant ethics board that reviewed the study protocol, and indicate whether they approved this research or granted a formal waiver of ethical approval<br>• Include an approval number if one was obtained<br>• If the study involved *non-human primates*, add *additional details* about animal welfare and steps taken to ameliorate suffering<br>• If anesthesia, euthanasia, or any kind of animal sacrifice is part of the study, include briefly which substances and/or methods were applied<br><br>**Field Research**<br><br>Include the following details if this study involves the collection of plant, animal, or other materials from a natural setting:<br>• Field permit number<br>• Name of the institution or relevant body that granted permission	
**Data Availability**<br><br>Authors are required to make all data underlying the findings described fully available, without restriction, and from the time of publication. PLOS allows rare	Yes - all data are fully available without restriction

exceptions to address legal and ethical concerns. See the PLOS Data Policy and FAQ for detailed information.<br><br>A Data Availability Statement describing where the data can be found is required at submission. Your answers to this question constitute the Data Availability Statement and **will be published in the article**, if accepted.<br><br>**Important:** Stating 'data available on request from the author' is not sufficient. If your data are only available upon request, select 'No' for the first question and explain your exceptional situation in the text box.<br><br>Do the authors confirm that all data underlying the findings described in their manuscript are fully available without restriction?	
**Describe where the data may be found in full sentences. If you are copying our sample text, replace any instances of XXX with the appropriate details.**<br><br>• If the data are **held or will be held in a public repository**, include URLs, accession numbers or DOIs. If this information will only be available after acceptance, indicate this by ticking the box below. For example: *All XXX files are available from the XXX database (accession number(s) XXX, XXX.*).<br>• If the data are all contained **within the manuscript and/or Supporting Information files**, enter the following: *All relevant data are within the manuscript and its Supporting Information files.*<br>• If neither of these applies but you are able to provide **details of access elsewhere**, with or without limitations, please do so. For example:<br><br>*Data cannot be shared publicly because of [XXX]. Data are available from the XXX Institutional Data Access / Ethics Committee (contact via XXX) for researchers who meet the criteria for*	The data set analyzed in this study is publicly available from the DHS website (https://dhsprogram.com/)

access to confidential data.<br><br>*The data underlying the results presented in the study are available from (include the name of the third party and contact information or URL).*<br>• This text is appropriate if the data are owned by a third party and authors do not have permission to share the data.<br><br>* typeset	
Additional data availability information:	

Powered by Editorial Manager® and ProduXion Manager® from Aries Systems Corporation

Dear Editor,

Please find attached our manuscript of research article for publication in **PLOS ONE entitled**: "**Trends and Disparities in Antenatal Care Utilization: A Multilevel Analysis of Four and Eight ANC Visits among Urban Women in Ethiopia**"

By: Work Dechassa [1], Wubegzier Mekonnen [1] Dessalegn Y. Melesse[3]

This study intended to examine trends and disparities of four plus and eight plus antenatal care utilization in urban Ethiopia between 2000 and 2019 using a multilevel analysis of Ethiopian demographic health survey data. Antenatal care is the gateway of the continuum of reproductive health care, offering a framework for critical healthcare functions such as complication prevention, screening, diagnosis, and health promotion. The manuscript hasn't been submitted in PLoS ONE and any other journal before.

In order to improve maternal health service utilization, specifically ANC4+ and ANC8+ and close the remaining gaps and disparities through policy implications, this research generally pursued to identify the trends and disparities of ANC4+ and ANC8+ utilization during critical time of women's life. Limited studies have used demographic health survey to describe the trends and disparities of ANC4+ and ANC8+ in urban setting. We think that our work, as presented in this manuscript, would be very helpful in reflecting insight on trends and disparities of ANC4+ and ANC8+ utilization in the urban of Ethiopia, which could be useful in creating effective interventions at community level and serve as a foundation for future research if it is published.

In case of any questions, please do not hesitate to contact us anytime.

Sincerely,

Worku Dechassa Heyi (on behalf of all authors)
Tel. +251912081108
E-mail:worku.dechassa@aau.edu.et

P. O. Box: 1176 Addis Ababa, Addis Ababa University

Manuscript

Click here to access/download;Manuscript;Manuscript_ANC48_2.docx

Trends and Disparities in Antenatal Care Utilization: A Multilevel Analysis of Four and Eight ANC Visits among Urban Women in Ethiopia

Work Dechassa [1*], Wubegzier Mekonnen [1] Dessalegn Y. Melesse[3]

[1]Department of Reproductive, Family and Population Health, School of Public Health, College of Health Sciences, Addis Ababa University, Ethiopia.

[2] Institute for Global Public Health, University of Manitoba, Winnipeg, Canada

***Corresponding author**

Email: worku.dechassa@aau.edu.et

Abstract

Introduction: Antenatal care is the gateway of the continuum of reproductive health care, offering a framework for critical healthcare functions such as complication prevention, screening, diagnosis, and health promotion.

Objective: This study examined disparities and trends of four plus and eight plus antenatal care utilization in urban Ethiopia between 2000 and 2019.

Methods: Datasets from the 2000, 2005, 2011, 2016, and 2019 Ethiopian Demographic and Health Surveys were analyzed using Stata version 16.1. The datasets were analyzed and disaggregated by wealth status, education, and region. Multilevel logistic regression analysis was used to identify factors associated with four plus and eight plus antenatal care utilization. The significance of variables was assessed using odds ratio(AOR) and 96% confidence interval at a significance level of $p<0.05$. Results: Four or more antenatal care(ANC4+) utilization increased from 43.1% in 2000 to 58.7% in 2019, which means that coverage increased by 15.6 percentage points over the two decades. Whereas, eight or more antenatal care (ANC8+) utilization decreased from 11.4% in 2000 to 3.6% in 2019, with a notable 7.8 percentage point decline across the survey years. The four or more antenatal care utilization among urban non-poor changed from 60.5% in 2000 to 81.8% in 2019. While the utilization among urban poor over 20 years changed from 37.8% in 2000 to 48.7% in 2019. Concerning the regional variation, Addis Ababa recorded the highest ANC4+ utilization, increasing from 72% in 2000 to 82% in 2019 a 10 percentage point increase. In contrast, ANC8+ utilization in the region declined sharply, from 38% in 2000 to 10% in 2019, marking a 28 percentage point decrease. The Somali region had the lowest ANC4+ utilization, though it increased from 9% in 2000 to 24% in 2019 a 15 percentage point increase. Meanwhile, Gambella had the lowest ANC8+ utilization, dropping from 5% in 2000 to 0% in 2019.

Wealth disparities were also pronounced; urban non-poor women had significantly higher odds of utilizing both ANC4+ (AOR: 2.43, 95% CI: 2.12–2.78) and ANC8+ (AOR: 3.02, 95% CI: 2.49–3.65) compared to urban poor women. Similarly, significant differences were observed by maternal education, with women with secondary or higher education more likely to utilize ANC services (ANC4+: AOR: 3.02, 95% CI: 2.54–3.59; ANC8+: AOR: 2.13, 95% CI: 1.63–2.78). Women aged 30-34 years had higher odds of utilizing ANC4+ (AOR: 2.89, 95% CI: 2.10–3.98) and ANC8+ (AOR: 2.22, 95% CI: 1.37–3.59). Additionally, media exposure at least once a week

was positively associated with increased utilization of ANC services, with women having 1.53 times higher odds of utilizing ANC4+ (AOR: 1.53, 95% CI: 1.28–1.83).

Conclusion: This study showed the significant regional, wealth, education disparities in ANC utilization in urban Ethiopia. Wealth status was the strongest predictor of ANC utilization, with urban non-poor women more likely to utilize ANC4+ and ANC8+ services. Maternal education, age, media exposure, and marital status also showed positive associations, while regional disparities persisted, with Addis Ababa outperforming other urban areas. To enhance ANC utilization, particularly among urban poor women, targeted intervention, media awareness creation, and educational initiatives should be prioritized.

Keywords: Trends, ANC4+, ANC8+, Utilization, Urban, poor, women in RH group, Ethiopia.

Introduction

Maternal health, as defined by the World Health Organization (WHO), is "the health of women during pregnancy, childbirth, and the postpartum period." To maximize the health and well-being of women and their unborn children, each stage of maternal care should be enjoyable (1). Maternal health services are crucial, as they can save millions of lives among women of reproductive age (2). However, about 800 women worldwide die daily from pregnancy or childbirth complications, with an estimated 287,00 deaths occurring in 2021 (3). Ninety-nine percent of maternal deaths take place in developing nations, while more than half of these fatalities take place in sub-Saharan Africa (SSA). Ethiopia is responsible for nearly 3.6% of maternal deaths in the world(4). The high rate of maternal deaths is due, in part, to unequal access to services for maternal health, and it emphasizes the wealth gap(2).

Low levels of maternal health service utilization, particularly in developing nations, are liable for the high rate of maternal mortality. Maternal health services utilization refers to how often pregnant women use antenatal care (ANC), delivery, and postnatal care services. Antenatal care is the gateway for reproductive health services, and it is a vital component of the reproductive healthcare continuum that offers a framework for critical healthcare functions such as complication prevention, screening, diagnosis, and health promotion. It is proven that ANC can save lives when it is utilized timely and adequately. Most importantly, ANC allows women to interact with and

receive support from families, communities, and other women during a crucial period in their lives(5).

Despite significant investments in maternal health services over the past 25 years, including prioritization within the national reproductive health strategy, utilization remains low in Ethiopia. This gap between service availability and utilization contributes to the country's persistent maternal health challenges(6). On the other hand, despite their demonstrated value in reducing obstetric risks(7,8), not all urban residents utilize ANC services (9). In line with this, it can be more difficult for low-income urban populations, especially those residing in slum areas and squatter settlements, to access quality maternal health services.

A study from Ghana indicates that compared to non-slum areas, maternal health service coverage is substantially lower in urban slums. Between 2018 and 2021, the attendance of antenatal care, in particular, has dramatically decreased in slum districts as compared to non-slum districts (10).

The sustainable development goal (SDG) era prioritizes health, in SDG target 3, focused on ensuring healthy lives and well-being for all at all ages. Achieving equitable, inclusive, and economically vibrant cities depends on the health of their populations. To improve health and attain universal health coverage (UHC), it is imperative to address the social determinants of health, especially in densely populated urban areas where maternal risks and inequities significantly impact social and economic outcomes. In many cities, some residents reap substantial benefits from urban living, while others experience no advantage at all. Cities in low and middle-income countries(LMICs) struggle to allocate limited resources due to rapid population growth, leading to the proliferation of slums and informal settlements that disproportionately affect mothers and children (11).

To lessen the significant and growing inequalities in urban areas, which are caused by an increasing number of urban poor people in Ethiopia, improved access to basic healthcare services is required. The Federal Ministry of Health (FMOH) developed the Urban Health Extension Program (UHEP) in 2009 to address the distinct and changing health issues the urban populations face(12).

Several studies on interventions for maternal and newborn babies have demonstrated that the provision of adequate ANC brings with it a positive impact on pregnancy outcomes, through early

diagnosis and appropriate management. About 39% of neonatal mortality is reduced due to the provision of adequate ANC to women in Sub-Saharan Africa (13–16).

The World Health Organization in its earlier guideline recommended that every pregnant woman with a normal pregnancy in middle and low-income countries attend at least four ANC visits, and the revised new guideline for safe pregnancy outcomes recommended women to attend eight or more ANC visits (5). However, many women in low-income countries, particularly in SSA, have limited access and low maternal health service utilization(17). Several studies have attempted to identify the factors associated with the low utilization of ANC4+ (16, 18–21). Age(16,18–22), Women's education, husbands' education, socioeconomic status (13,15,16,18–22), Women's employment, birth order, media exposure (13,15,16,18–23), and place of residence(19,22,23) are among the factors associated with underutilization of ANC. The factors attributed to less than the recommended number of ANC utilization are quite substantial across regions and subpopulations (18,24).

Due to the paucity of information in the area, this study aimed to examine disparities and trends of four plus and eight plus ANC visit utilization in urban areas of Ethiopia, using the Ethiopian Demographic and Health Survey (EDHS) series from 2000 to 2019.

Methods and materials

The Ethiopian Demographic and Health Survey conducted in 2000, 2005, 2011, 2016, and 2019(Mini DHS) based on nationally representative samples of women aged 15–49 years that provide estimates at the national and regional levels is analyzed. EDHS covers a variety of indicators, including sexual, reproductive, and maternal health service utilization among women aged 15-49 years. Study participants are selected through a two-stage cluster sampling method that is stratified by type of residence (urban or rural) and study regions which constituted delineated homogeneous enumeration areas(EAs) or clusters. A sample of EAs is selected using probability proportional to size within each stratum (urban or rural) from the list of predefined EAs. Subsequently, members of households within the selected EAs are surveyed using a systematic random sampling approach, focusing on women aged 15 to 49 years and men aged 15 to 59 years. Individual records (IR files) on women aged 15 to 49 years were used for this study. Details on the EDHS study methods are published by Ethiopia's Central Statistics Agency(25–28).

Variables and measurements

Receipt of ANC was defined as a woman reporting any facility-based ANC visit during the pregnancy of the most recent live birth. The primary outcome variables were categorized into: women who had at least four ANC visits (categorized as "yes -those who had four or more ANC visits" or "no -those who had no ANC visit or those who had one to three visits") and women who had at least eight ANC visit (categorized as "yes-those who had eight or more ANC visits" or "no-those who hand no ANC visit or those who had one to seven visits) for which trends over twenty years have been assessed. It was calculated as the percentage of pregnant women who attended four or more or eight or more ANC visits in a given year of the total number of pregnant women aged 15–49 years surveyed in the same year. Trends of ANC service utilization were stratified by wealth status, education status, and region. Wealth status was computed using the country-specific and urban/rural-adjusted wealth quintiles variable (v190a), designed by the DHS team (29). Then, based on the median wealth index categorized into urban poor and urban non-poor. Maternal educational status was categorized into three categories: no education, primary education, and secondary and higher education. The sub-national regions include nine regions and two city administrations; no sequence was shown in sub-national region segregation.

Multilevel Regression Analysis (MLRA)

The study used multilevel analysis methods to account for the hierarchical nature of the DHS data by categorizing the outcome variable into binary response. The hierarchical structure of the data follows an individual's (level 1) who are nested within communities (level 2) S1 Figure 1. The level 1 model represents the relationships among the individual-level variables and the outcome variable, while the level 2 model examines the influence of community-level factors on the outcome variable. The target population was all women aged 15–49 years in randomly selected households across urban areas in nine regions and two administrative cities. All urban women aged 15–49 years considered in the series of EDHS were eligible for this study. A total of 6366 urbanite women participated in the five rounds of the EDHS survey were considered for Antenatal care utilization analysis. The Stata Software version 16.1 was used for the analysis. and 95% CI was computed to measure statistical significance. Finally, *P* values less than 0.05 were used to declare the significance level.

According to the current WHO recommendation, a woman is said to utilize optimal ANC if she has eight or more ANC visits at a health facility, but, it is challenging to achieve the eight visit, while ANC4 remains sub-optimal(5). Accordingly, this study examined both ANC4 and ANC8 utilization as reported across the survey period. The response variable (Yic), four or more ANC, eight or more ANC utilization, is a binary outcome variable and is categorized as:

$$Yic = \begin{cases} 1, \text{ if a woman has 4 or more ANC visits, eight or more ANC} \\ 0, \text{ if less than 4 or no ANC visits, less than eight or no ANC} \end{cases}$$

The null model is used to decompose the total variation into women-level like (education, wealth status, age, marital status, family size, child birth order, working status) and cluster-level (region, religion, year) variations. In the random intercept model, the intercept term is the only random effect; the group differs concerning the average value of the response variable, and the slope is assumed to be fixed across all the regions. The random coefficient model allows the explanatory variables to have different effects for different regions. The functional forms were described below

$$\text{Logit}(\pi ij) = \beta o + uoj \qquad \text{Null model}$$

$$\text{Logit}(\pi ij) = \beta o + \sum_{h=1}^{p} \beta hXhij + uoj \qquad \text{Random intercept model}$$

$$\text{Logit}(\pi ij) = \beta o + \sum_{h=1}^{p} \beta hXhij + \sum_{h=1}^{p} \beta uhjXhij + uoj \qquad \text{Random coefficient model}$$

Where: i = 1, 2, 3, ...:n_j and j =1, 2, . . . ; 11, π_{ij} is the probability of attending four or more ANC for i^{th} women in j^{th} region, β0 is the population average of the transformed probabilities, β_h, h= 1; 2; . . . ; p are fixed slope for p explanatory variables, X_{hij}; h= 1; 2; . . . ; p are explanatory variables at women level and regional level; U_{hj} ; h= 1; 2; . . . ; p are random slope for p explanatory variables and u_{0j} is the random effect. u_{0j} ND(0; δ_u^2), $\binom{uoj}{uhj}$MND (0_{p+1}; $Ɛ_{p+1}$). Furthermore, βo+ $\sum_{h=1}^{p} \beta hXhij$ are assumed to be fixed, and $\sum_{h=1}^{p} \beta uhjXhij + uoj$ are assumed to be random. The Inter-class Correlation Coefficient (ICC) under the null model is computed as: ICC = $\frac{\sigma^2_u}{\sigma^2_u+\sigma^2_e}$ where σ_e^2 is women-level variance, which is assumed to be fixed to $\frac{\pi^2}{3}$= 3:29, and σ_u^2 is regional-level variance, which is obtained from the analysis. The model-building strategy is exploratory and aims to assess how the inclusion of variables and their interactions impacts model

improvement. The model-building process was described in S2 Figure 2. The parameters of the model were estimated by the maximum likelihood method. Model selection was done using Akaike's information criteria (AIC) and Bayesian information criteria(BIC).

Four models having variables of interest were fitted. Model 1: It is the null model, used to check the variability among the communities without inserting exposure variable. It's the first step in MLRA used to provide evidence of whether the data has justifiable evidence to assess the random effects at the community level. Model 2: It is a multivariate model adjustment for individual-level variables that were significant at the bivariate MLRA level. It was built by a stepwise regression analysis technique, which enables the removal of independent variables that become insignificant during the insertion of every other independent variable into the model. Hence, independent variables that were significant in model 2 (i.e., individual-level variables model adjustment) were considered candidates for the final model. Model 3: It is a multivariate model adjustment for community-level variables that were significant at the bivariate MLRA level. All independent variables that were statistically significant in model 3 (community-level variables model adjustment) were included in the final model as potential candidates.

Model 4: It is a multivariate MLRA model adjustment of the outcome variable against predictor variables, which were statistically significant either at the individual or community level model adjustments. In this model, Variables were categorized as community-level or individual-level factors. Community-level factors were survey year, religion, place of previous residence and region; individual-level factors were wealth status, maternal education, maternal age, birth order, partner's educational level, sex of the household head, marital status, husband/partner's educational level, family size, number of living children, exposure to mass media and Working Status Parameter estimations

The measures of association (fixed-effects) estimate the associations between the likelihood of ANC4+/ANC8+ utilization and various explanatory variables measured using an adjusted Odds Ratio (AOR) with their 95 % Confidence Intervals (CIs). The measures of variation (random effects) were reported as the Intra-community correlation coefficient (ICC), which is the percentage variance explained by the higher level (community level variables). Change in Community Variance (CV), expresses the change in the community-level variance between the empty model (Model 1) and the subsequent models.

Multicollinearity

The presence of multicollinearity was checked among independent variables using the Variance Inflation Factor (VIF) at the cut-off point of 10. Factors containing a VIF value of less than 10 indicate the absence of multicollinearity. All the variables included in the analysis had a VIF of less than three, indicating the absence of multicollinearity.

Fitness of the Model

Akakie Information Criterion (AIC) was used to estimate the goodness of fit of the adjusted final model in comparison to the preceding models (individual and community level model adjustments). The AIC value for each subsequent model was compared, and the model with the lowest value was considered to be the best-fit model and identify determinant factors of ANC4+ and ANC8+ service utilization.

Results

Sociodemographic characteristics of the study participants

The pooled mean age of study participants from all the survey year was 28.56±(0.14), Concerning the age distribution of the study participants from all the survey years, the highest proportions (34.8%) of women were in the age group of 25-29 years followed by (19.2%) aged between 30-34 years. Among the study participants 86.5% of them were married. Concerning the education of the study sample from all the survey years, 34.0% of them attended primary education. Nearly half (46.8%) of husbands/partners attended a secondary or higher education level. Over half of the study participants were Orthodox Christians follower (56.9%). Nearly three-quarter (73.9%) of the households were headed by males, and 20.4% of them were between the ages of 30-34. Approximately 66.3% of the study sample belonged to a low wealth status, and 59.5% of the study sample were not working during the survey period (Table 1).

Table 1: Socio-demographic characteristics of the study sample from all the survey years, Ethiopian Demographic Health Survey 2000-2019 (n=6366)

Variables	Categories	Frequency	Percentage
Age of women in a complete year	15-19	287	4.2
	20-24	1426	21.9
	25-29	2156	34.8
	30-34	1282	19.2
	35-39	860	13.0

	40-44	280	5.3
	45-49	75	1.6
Marital status	Never married	139	2.0
	Married/Cohabitation	5455	86.5
	Divorced/Separated/Widowed	772	11.5
Respondents' Highest level of education	No education	1980	32.5
	Primary	2023	34.0
	Secondary/higher	2363	33.5
Sex of household head	Male	4461	73.9
	Female	1905	26.1
Age of household head in a complete year	15-19	35	0.4
	24-24	387	6.1
	25-29	1149	19.5
	30-34	1302	20.5
	35-39	1218	18.4
	40-44	813	12.9
	45-49	490	8.1
	50+	972	14.1
Husband/partner's educational level	No formal education	1035	22.1
	Primary	1430	31.1
	Secondary/higher	2613	46.8
Religion	Orthodox	3205	56.4
	Muslim	2291	24.4
	Protestant	806	18.1
	Others	64	1.1
Wealth status	Urban poor	3418	66.5
	Urban non-poor	2948	33.5
Working status	Not working	3301	59.5
	Working	2061	40.5

Exposure to Media Outlet

The analysis of media exposure among the study sample from the EDHS 2000 to 2016 reveals notable patterns in the percentage of newspaper/magazine reading, listening radio, and watching television. Over the years, watching TV show the most significant increase, more than tripling over the 15 years (12.7% to 58.4%). The least used media outlet was reading newspaper/magazine changing from 5.0% to 8.7% over 15 years. On the other hand, listening to the radio slightly remain similar over the 15 years (30.3% to 32.7%. When considering all forms of media outlet, slightly increased over time from 10.1% to 28.5% as shown in S3 Figure 3.

Reproductive Health Characteristics and Service Utilization

The reproductive health characteristics of the study sample using data from all survey rounds(pooled) of EDHS 2000 to 2019 revealed that a significant proportion of respondents, 44.8%, had their first birth between the ages of 15 and 19. Approximately 40.1% of women initiated their first antenatal care (ANC) visit during the second or third month of pregnancy, while a smaller percentage, 4.6%, sought ANC within the first month. More than half of the respondents, 58.9%, delivered in health facilities; however, a substantial 41.1% of births still occurred at home. Regarding the sex of the children, the distribution between male and female children was relatively balanced, with males constituting 51.0% and females 49.0%. Additionally, over half of the respondents, 55.4%, reported having one or two living children(Table 2)

Table 2: Reproductive health characteristics of the study sample from all survey round(pooled), EDHS 2000-2019

**Variables**	**Categories**	**Frequency**	**Percentage**
Age of respondent at first birth	9-14	348	6.6
	15-19	2694	44.8
	20-24	2317	35.6
	25-29	774	10.4
	30+	236	2.6
Timing of first ANC check up	In the 1st one month	347	4.6
	2-3months	2,379	40.1
	4months	1,220	24.3

	5-9months	1,314	31.1
Place of Delivery	Home	2,043	41.1
	Health facility	4,326	58.9
Sex of child	Male	3260	51.0
	Female	3109	49.0
Number of living children	No child	117	1.9
	1-2 child	3783	55.4
	3+child	2469	42.7

Regional Disparities and Trends in Antenatal Care Utilization

The data reveals key trends in antenatal care (ANC) across urban Ethiopia from 2000 to 2019, highlighting improved ANC4+ (four or more visits) coverage in most regions through 2016, followed by a slight decline to 59% by 2019. Addis Ababa consistently led, peaking at 89% in 2016, while regions like Tigray and Benishangul also showed strong gains. In contrast, Somali and Afar remained low, with Somali dropping to 24% in 2019. ANC8+ (eight or more visits) utilization remained low throughout, rarely surpassing 30%. Addis Ababa demonstrated the highest early rates (38–42%) but dropped to 10% by 2019. Dire Dawa maintained relatively better coverage, while most other regions stayed below 10%, with national ANC8+ utilization falling from 15% in 2005 to just 4% in 2019 S4 Figure 4.

Trends of antenatal care utilization in urban Ethiopia

Four or more antenatal care utilization was increased from 43.1% (95% CI; 38.3 - 48.5) in (2000), to 54.6% (95% CI; 49.8-59.2) in (2005) with an annual change of 5.3%, but, changed from 54.6% in 2005 to 45.5% in 2011 with an annual change of -2.8%. ANC4+ utilization during the last 20 years changed from 43.1% (95% CI: 38.3 - 48.5) in 2000 to 58.7% (95% CI: 52.9-64.2) in 2019, with an average annual change of 0.82%. Eight or more ANC utilization was changed from 11.4%(95% CI; 8.9, 14.8) in (2000) to 15.4% (95%; 12.7-18.5%) in 2005, with annual change of 7.0%, but changed from 15.4% in 2005 to 9.8% in 2011 with annual change of --6.1%. Eight or more ANC utilization during the last 20 years changed from 11.4% (95% CI; 8.9 - 14.8) in 2000, to 3.6% (95% CI; 2.4-5.4) in 2019, with an average annual change of -0.4% (Table 3)

Table 3: The trends of ANC4+ and ANC8+ utilization in Urban Ethiopia from EDHS 2000 -2019

Year	ANC4+ utilization at national level(urban)	Annual % changes in ANC4+	95%CI	ANC8+ utilization at national level(urban)	Annual % changes in ANC8+	95%CI
2000	43.1%	-	38.3%- 48.5%	11.4%	-	8.9% - 14.8%
2005	54.6%	5.3%	49.8%- 59.2%	15.4%	7.0	12.7% -18.5%
2011	45.5%	-2.8%	41.2%- 49.9%	9.8%	-6.1	7.9% - 12.2%
2016	62.7%	7.4%	57.9%- 67.2%	10.4%	1.2	8.4% - 12.6%
2019	58.7%	-2.1%	52.9%- 64.2%	3.6%	-21.9	2.4% - 5.4%
	Average annual change	0.8			-0.4	

As shown in S5 Figure 5, From 2000 to 2019, ANC4+ utilization increased among both urban poor and urban non-poor women, with the urban poor rising from 37.8% to 48.7% (a 28.8% increase), and the urban non-poor from 60.5% to 81.8% (a 35.2% increase). In contrast, ANC8+ utilization declined significantly for both groups: among the urban poor, it dropped from 7.8% to just 0.6% (a 92.3% decrease), while among the urban non-poor, it fell from 23.3% to 10.5% (a 54.9% decrease.

Maternal education serves as an important equity stratifier, highlighting both absolute and relative differences in antenatal care (ANC) utilization. Among women with secondary or higher education, the proportion reporting ANC4+ visits increased from 60.9% in 2000 to 81.3% in 2019, representing an overall increase of 33.5%. In contrast, ANC8+ utilization in this group declined significantly from 18.7% to 6.2% over the same period, a 66.8% decrease. For women with no formal education, ANC4+ utilization also showed improvement, rising from 25.1% in 2000 to 43.0% in 2019, an overall increase of 71.3%. However, ANC8+ coverage in this group dropped from 3.5% to 1.6%, reflecting a 54.3% decrease S6 Figure 6.

Factors Associated with ANC4+ and ANC8+ utilization

The factors that are significantly associated with ANC4+ utilization from pooled data of the EDHS 2000 to 2019, were women's age, marital status, pregnant women’s education, partner/husband’s educational level, wealth status, family size, mass media exposure, and childbirth order ($p<0.05$). Women aged 30-34 years showed the highest ANC4+ utilization with significant differences across age groups ($p < 0.002$). The proportion of ANC4+ utilization was higher among married women as compared to unmarried women. The proportion of respondents reporting four or more antenatal utilization increased with higher educational attainment (71.4% for those attended secondary or higher education as compared to 33.1% with no education. Similar patterns were observed for husband/partner education; women whose partners had secondary or higher education reported a higher proportion of ANC4+ utilization. Similarly, at least once a week media exposure was associated with an increased in the four or more antenatal care utilization(P-value< 0.000)(Table 4).

In urban settings, poor women had significantly lower ANC4+ utilizations (41.9%) than urban non-poor women (74.5%) ($p < 0.001$). Regarding birth order, first mother had the highest ANC4+ utilization (57.9%), with significant variations across different birth orders ($p < 0.000$). There were significant regional disparities that existed in ANC4+ utilization (P-value< 0.000), with Addis Ababa reporting the highest (83.0%) and the Somali region reporting the lowest (22.0%). There was a noticeable trend over the years, with highest ANC4+ utilization in 2016 , with significant differences across years at (P-value< 0.000) (Table 4).

Factors Associated with ANC8+ utilization

The factors that are significantly associated with ANC8+ utilization were the age of women, pregnant women’s education, partner/husband’s educational level, wealth status, mass media exposure, working status, and childbirth order ($p<0.001$). Women aged 30-34 years showed the highest (11.8%) ANC8+ utilization with significant differences across age groups ($p < 0.002$). The proportion of respondents reporting $\geq$8 antenatal care utilization increased with higher educational attainment. Similar patterns were observed for partner /husband’s education; women whose partner had secondary or higher education reported a higher (16.4%) proportion of $\geq$8 ANC utilization. Similarly, at least once a week media exposure was associated with an increased (20.7%) in the eight or more antenatal care utilization(P-value< 0.000)(Table 4).

In urban settings, poor women had a significantly lower proportion of ANC8+ utilization (4.4%) than urban non-poor women (20.3%) (p< 0.000). working status also mattered, as currently working women showed higher (13.0)8 ANC8+ utilization than non-working women (8.2%) (p < 0.000). Regarding birth order, first time and two-three times mothers had the highest (11.1%) ANC8+ utilization, with significant variations across different birth orders (p < 0.001). There were significant regional disparities that exist in ANC8+ utilization (P-value< 0.000), with Addis Ababa reporting 31.1%. There was a noticeable trend over the years, with decreasing ANC8+ utilization from 2000 to 2019, the highest (15.4) ANC8+ utilization observed in 2005with significant differences across years(Table 4).

Table 4: Differences in ANC4+ and ANC8+ utilization status across different categories of exposure variables and their Chi-square test of association in Urban Ethiopia from pooled data of EDHS 2000 to 2019

Variables	Categories	**ANC4+** No N(%)	**ANC4+** Yes N(%)	Chi-Square (p-value)	**ANC8+** No N(%)	**ANC8+** Yes N(%)	Chi-Square (p-value)
Sex of household head	Male	2173(46.2)	2533(53.8)	2.5(0.111)	4271(90.7)	435(9.3)	2.5(0.115)
	Female	832(50.1)	828(49.9)		1476 (88.9)	184(11.1)	
Age of the mother	15-19	160(59.7)	109(40.3)	3.5(0.002)	257(95.4)	12(4.6)	3.6(0.002)
	24-24	673(48.2)	723(51.8)		1293(92.6)	104(7.4)	
	25-29	1026(46.4)	1187(53.6)		1966(88.9)	247(11.1)	
	30-34	507(41.5)	714(58.5)		1077(88.2)	144(11.8)	
	35-39	372(44.8)	457(55.2)		750(90.6)	78(9.4)	
	40-44	198(58.9)	138(41.1)		305(90.8)	31(9.2)	
	45-49	68(66.9)	34(33.1)		99(97.3)	3(2.7)	
Marital status	Never married	79(62.3)	48(37.7)	16.9(0.000)	107(84.4)	20(15.6)	1.5(0.229)
	Married/Cohabitation	2481(45.0)	3026(55.0)		4976(90.4)	531(9.6)	
	Divorced/Separated/ Widowed	445(60.7)	287(39.3)		664(90.7)	68(9.3)	
Respondents' level of education	No education	1384(66.9)	686(33.1)	91.9(0.000)	2003(96.8)	67(3.2)	57.1(0.000)
	Primary	1012(46.7)	1155(53.3)		1964(90.6)	203(9.4)	
	Secondary/higher	608(28.6)	1521(71.4)		1780(83.6)	349(16.4)	
Husband/partner 's level of educational	No education	1445(54.4)	1212(45.6)	25.5(0.000)	2544(95.8)	113(4.2)	59.0(0.000)
	Primary	749(50.6)	731(49.4)		1355(91.5)	125(8.5)	
	Secondary/higher	811(36.4)	1418(63.6)		1848(82.9)	381(17.1)	
Wealth status	Urban poor	2461(58.1)	1771(41.9)	205.4(0.000)	4045(95.6)	186(4.4)	169.1(0.000)
	Urban non-poor	544(25.5)	1590(74.5)		1702(79.7)	433(20.3)	
Family size	1-5	1738(44.8)	2145(55.2)	7.2(0.007)	3505(90.3)	377(9.7)	0.005(0.98)

	6+	1267(51.0)	1216(49.0)		2241(90.3)	243(9.7)	
Exposure to Mass Media	Not at least once a week	2690(50.5)	2636(49.5)	41.8(0.000)	4922(92.4)	404(7.6)	95.2(0.000)
	At least once a week	315(30.2)	725(69.8)		825(79.3)	215(20.7)	
Working status	Currently not working	2068(47.6)	2279(52.4)	0.2(0.627)	3990(91.8)	356(8.2)	18.4(0.000)
	Currently working	937(46.4)	1082(53.6)		1757(87.0)	263(13.0)	
Birth Order	1	808(42.1)	1109(57.9)	14.3(0.000)	1707(89.0)	210(11.0)	5.4(0.001)
	2-3	1129(44.3)	1420(55.7)		2265(88.9)	284(11.1)	
	4-5	489(48.3)	522(51.7)		930(92.1)	81(7.9)	
	6+	579(65.1)	310(34.9)		845(95.0)	44(4.9)	
Religion	Orthodox	1494(41.6)	2097(58.4)	11.8(0.000)	3146(87.6)	445(12.4)	14.6(0.000)
	Muslim	836(53.9)	715(46.1)		1456(93.9)	95(6.1)	
	Protestant	622(53.9)	532(46.1)		1075(93.2)	78(6.8)	
	Others	52(74.4)	18(25.6)		68(98.4)	1(1.6)	
Region	Addis Ababa	182(17.0)	884(83.0)	48.2(0.000)	733(68.9)	332(31.1	52.1(0.000)
	Afar	50(58.1)	36(41.9)		81(94.8)	4(5.2)	
	Amhara	725(55.2)	588(44.8)		1253(95.4)	60(4.6)	
	Benishangul Gumuz	30(46.9)	34(53.1)		62(96.1)	3(3.9)	
	Dire Dawa	34(35.8)	62(64.2)		83(85.8)	14(14.2)	
	Gambella	26(53.5)	23(46.5)		48(98.3)	1(1.7)	
	Harari	19(40.9)	27(59.1)		39(84.6)	7(15.4)	
	Oromia	998(55.5)	800(44.5)		1706(94.9)	92(5.1)	
	SNNPR	504(54.1)	428(45.9)		890(95.5)	42(4.5)	
	Somali	257(78.0)	73(22.0)		322(97.5)	8(2.5)	
	Tigray	180(30.7)	406(69.3)		529(90.3)	57(9.7)	
Survey Year	2000	686(56.2)	534(43.8)	11.6(0.000)	1075(88.1)	145(11.9)	12.6(0.000)
	2005	388(45.5)	466(54.5)		723(84.6)	131(15.4)	
	2011	872(54.5)	729(45.5)		1444(90.2)	158(9.8)	
	2016	487(37.3)	819(62.7)		1171(89.7)	135(10.3)	
	2019	571(41.3)	813(58.7)		1334(96.4)	50(3.6)	

Multilevel analysis of Antenatal care utilization from pooled data of 2000 to 2019 EDHS

Multilevel binary logistic regression was carried out to identify factors associated with four plus and eight plus visits of antenatal care utilization (ANC4+ and ANC8+). Of the community-level factors examined, religion and geographic area(region) were statistically significantly associated with ANC4+ and ANC8+ utilization in final model and survey year was also considered as level

two variable associated with ANC4+ and ANC8+ utilization. Of the individual-level factors, age of respondent, education of the pregnant woman, partner/husband's educational level and, wealth status showed statistically significant associations with ANC4+ and ANC8+ service utilization in the final model. On the other hand, size of the family, marital status, birth order of the child and exposure to mass media showed statistically significant association with ANC4+ service utilization.

Women aged 30-34 had over 2 times the odds of attending ANC4+ (OR: 2.22, 95% CI: 1.60–3.08) compared to 15-19 years, with 40-44 group reaching AOR 3.3(95% CI: 1.50-3.58). In contrast, women in age group 45-49 years did not demonstrate statistically significant differences in ANC4+ utilization. With regard to ANC8+ utilization, women aged 30–34 years had 1.2 times higher odds (AOR: 1.77, 95% CI: 1.08–2.90) compared to 15-19 years, with 40-44 age group had 2 times higher odds (AOR: 2.11, 95% CI: 1.12-3.97) of ANC8+ utilization compared to 15-19 years' group.

Women who attained secondary and higher educational levels had 2.6 times higher odds of utilizing ANC4+ service as compared to women who haven't been in formal education (OR:2.62, 95% CI: 2.19-3.14). For ANC8+, the odds of utilizing ANC8+ were 2 times (AOR: 2.13, 95% CI: 1.63–2.78) higher for those who attended secondary and higher educational level. Similarly, women whose husband/partner attained secondary and higher educational level had 1.8 times (OR: 1.81, 95% CI: 1.50-2.19) and 1.6 times (OR: 1.56, 95% CI: 1.20-2.02) higher odds of utilizing ANC4+ and ANC8+ services as compared to women whose husband/partner had no formal education, respectively.

Urban non-poor women had substantially higher odds of both ANC4+ (AOR:1.65, 95% CI: 1.42 – 1.90) and ANC8+ (AOR: 1.96, 95% CI: 1.60-2.40) compared to their urban poor counterparts. On the other hand, mass media utilization was associated with ANC4+ utilization. Women who exposed mass media at least once a week had 1.5 times higher odds of utilizing ANC4+ service as compared to women who haven't been exposed to media at least once a week (OR:1.52, 95% CI: 1.27-2.82). Furthermore, mothers who had first birth order were 1.55 times (OR: 1.55, 95% CI: 1.16–2.07) more likely to utilize ANC4 as compared to reference group. Women from smaller families (1–5 members) had 21% higher odds of ANC4 utilization as compared to those from larger families (6+ members) (OR: 1.21, 95% CI: 1.06–1.40). Concerning marital status,

married or cohabiting women were two times (OR: 2.0, 95% CI: 1.31–3.07) more likely to utilize ANC4+ as compared to never-married women.

After controlling for other variables, the odds of a woman in the Somali region utilizing four or more antenatal care were reduced by 88% than a woman living in Addis Ababa (OR: 0.12, 95% CI: (0.09 – 0.17). On the other hand, the odds of a woman in the Gambella region utilizing four or more antenatal care were reduced by a dramatic 96% (AOR: 0.04, 95% CI: 0.02 - 0.10). Furthermore, Women who identify as Protestant (AOR: 0.72, 95% CI: 0.58–0.90) and those belonging to other religions (AOR: 0.52, 95% CI: 0.29–0.93) had significantly lower odds of attending at least four antenatal care (ANC) visits compared to Orthodox women. Additionally, Muslim women were less likely to complete eight or more ANC visits (AOR: 0.80, 95% CI: 0.65–0.99).

Regarding the survey year, by 2016, the odds of ANC4+ utilization had nearly doubled to an AOR of 1.97 and peaked at AOR of 2.73 in 2019, indicating steady progress in ANC uptake, while on the other hand, the odds of ANC8+ utilization were significantly lower in both 2016 (AOR = 0.66) and 2019 (AOR = 0.55) compared to the year 2000(Table 5).

Table 5: Multilevel Logistic regression results of individual and community level factors associated with ANC4+ service utilization among women aged 15- 49 years in urban Ethiopia from pooled 2000-2019 EDHS data by socioeconomic and demographic subgroups, EDHS 2000- to 2019

		ANC4+				ANC8			
Variable	Categories	Null Model	Model I AOR(95% CI)	Model II AOR(95% CI)	Model III AOR(95% CI)	Null Model	Model I AOR(95% CI)	Model II AOR(95% CI)	Model III AOR(95% CI)
Individual-level variables									
Age of women	15-19	-	1	-	1	-	1	-	1
	20-24	-	1.55(1.16 –2.07)*	-	1.43(1.08-1.92)*	-	1.22(0.77 – 1.95)	-	1.17(0.73-1.87)
	25-29	-	2.15(1.60 –2.88)*	-	1.79(1.33-2.41)*	-	1.66(1.05 –2.64)*	-	1.45(0.91-2.31)
	30-34	-	2.89(2.10 –3.98)*	-	2.22(1.60-3.08)*	-	2.22(1.37 –3.59)*	-	1.77(1.08-2.90)*
	35-39	-	3.03(2.15 –4.27)*	-	2.18(1.54-3.10)*	-	2.12(1.27 –3.54)*	-	1.56(0.93-2.64)
	40-44		3.18(2.07 –4.87)*		3.32(1.50-3.58)*		2.79(1.49 –5.19)*		2.11(1.12-3.97)*
	45-49		2.00(1.06 –3.78)*		1.35(0.71-2.56)		2.08(0.76– 5.73)		1.62(0.58-4.54)
Pregnant Women's Education	No Education	-	1	-	1	-	1	-	
	Primary	-	1.85(1.59 –2.15)*	-	1.57(1.35-1.84)*	-	1.46(1.13– 1.88)*	-	1.49(1.15-1.93)*
	Secondary/Higher	-	3.02(2.54 –3.59)*	-	2.62(2.19-3.14)*	-	1.99(1.54– 2.58)*	-	2.13(1.63-2.78)*
Husband/partner's education	No Education	-	1		1	-	1		1
	Primary	-	1.26(1.06-1.48)*		1.48(1.23-1.79)*	-	1.43(1.11-1.83)*		1.08(0.82-1.44)
	Secondary/Higher	-	1.33(1.13- 1.56)*		1.81(1.50-2.19)*	-	1.91(1.54- 2.36)*		1.56(1.20-2.02)*
Birth order	1	-	2.34(1.77 – 3.10)*	-	1.55(1.16 – 2.07)*	-	2.06(1.38 – 3.08)*	-	1.45(0.95– 2.21)
	2-3	-	1.79(1.40 –2.28)*	-	1.32 (1.03 1.70)*	-	1.73(1.19 –2.51)*	-	1.39(0.94–2.07)
	4-5	-	1.46(1.16 –1.84)*	-	1.25(0.98 – 1.58)	-	1.55(1.05 –2.29)*	-	1.43(0.96 –2.14)
	6+	-	1	-	1	-	1	-	1
Family size	1-5	-	1.27(1.10– 1.45)*		1.21(1.06 – .40)*		NI		
	6+		1		1				
Marital status	Never married	-	1	-	1		NI		
	Married/Cohabitation	-	1.96(1.30 –2.96)*	-	2.00(1.31-3.07)*				
	Divorced/Separated/Widowed	-	1.26(0.82– 1.93)	-	1.28(0.82-1.98)				
Wealth status	Urban poor	-	1	-	1	-	1	-	1
	Urban non-poor	-	2.43(2.12 –2.78)*	-	1.65(1.42 –1.90)*	-	3.02(2.49 –3.65)*	-	1.96(1.60 –2.40)*
Working status	Currently not working		NI				1		1
	Currently working						1.05(.89-1.24)		1.06(0.89-1.25)
Media exposure	Not at least once a week	-	1	-	1	-	1	-	1

	At least once a week	-	1.53(1.28 –1.83)*	-	1.52(1.27 – .82)*	-	1.31(1.09 –1.58)*	-	1.12(0.93 -1.35)
Community level variables									
Religion	Orthodox	1		1	1	1		1	1
	Muslim	-	-	0.57(0.49-0.66)*	0.87(0.74-1.02)	-	-	0.54(0.44-0.66)*	0.80(0.65-0.99)*
	Protestant	-	-	0.73(0.60-0.91)*	0.72(0.58-0.90)*	-	-	1.12(0.85-1.47)	1.08(0.82-1.44)
	Others	-	-	0.58(0.34-1.01)	0.52(0.29-0.93)*	-	-	0.47(0.16-1.42)	0.38(0.12-1.18)
Region	Addis Ababa	-	-	1	1	-	-	1	1
	Afar	-	-	0.17(0.12-0.24)*	0.25(0.18 – 0.35)*	-	-	0.19(0.11-0.32)*	0.30(0.17 - 0.51)*
	Amhara	-	-	0.17(0.13-0.23)*	0.27(0.20 – 0.36)*	-	-	0.11(0.06-0.18)*	0.17(0.10 - 0.29)*
	Ben Gumz	-	-	0.22(0.15-0.32)*	0.39(0.27 – 0.56)*	-	-	0.09(0.04-0.18)*	0.17(0.08 - 0.35)*
	Dire Dawa	-	-	0.43(0.35-0.54)*	0.51(0.40 – 0.64)*	-	-	0.41(0.32-0.53)*	0.50(0.39- 0.64)*
	Gambella	-	-	0.20(0.15-0.27)*	0.31(0.23 – 0.42)*	-	-	0.03(0.01-0.06)*	0.04(0.02 - 0.10)*
	Harari	-	-	0.34(0.27-0.43)*	0.31(0.24 – 0.39)*	-	-	0.46(0.35-0.59)*	0.43(0.33- 0.56)*
	Oromia	-	-	0.18(0.13-0.23)*	0.24(0.18 – 0.32)*	-	-	0.12(0.07-0.18)*	0.17(0.11 -0.28)*
	SNNPR	-	-	0.20(0.15-0.28)*	0.31(0.22 – 0.43)*	-	-	0.12(0.07-0.20)*	0.21(0.12 -0.35)*
	Somali	-	-	0.06(0.04-0.08)*	0.12(0.09 – 0.17)*	-	-	0.08(0.04-0.14)*	0.14(0.08- 0.27)*
	Tigray	-	-	0.43(0.33-0.58)*	0.65(0.49 – 0.87)*	-	-	0.22(0.16-0.32)*	0.31(0.22-0.45)*
Survey Year	2000	-	-	1	1	-	-	1	1
	2005	-	-	1.62(1.32-2.00)*	1.38(1.13-1.70)*	-	-	1.26(0.98- 1.63)	1.03(0.79-1.34)
	2011	-	-	1.54(1.27-1.87)*	1.25(1.03-1.52)*	-	-	1.30(1.02- 1.66)*	1.14(0.88-1.47)
	2016	-	-	2.54(2.09-3.09)*	1.97(1.62 – 2.41)*	-	-	0.86(0.67- 1.10)	0.66(0.50 – 0.85)*
	2019	-	-	2.15(1.72-2.70)*	2.73(2.11-3.54)*	-	-	0.52(0.38- 0.72)*	0.55(0.37- 0.82)*
	Intercept	1.75(1.59 – 1.94)*	0.06(0.04 –0.10)*	3.70(3.07-4.47)*	0.18(0.10 – 0.32)*	0.14(0.12 –0.16)*	0.01(0.01 – 0.02)*	0.05(0.41-0.62)*	0.07(0.03 – 0.13)*

Note **: indicates significance level at P-value<0.05, NI: Variable not included the model

Table 6: Model Diagnostics and Fit Indices of ANC4+ and ANC8+Service utilization among women aged 15-49 years in urban Ethiopia from pooled EDHS 2000 to 2019

**Model fitness summary for ANC4+ analysis**					**Model fitness summary for ANC8+ analysis**			
Model Evaluation Metrics	Null Model	Model I	Model II	Model III	Null Model	Model I	Model II	Model III Final
Random effect result								
ICC	18.30%	8.60%	6.80%	3.30%	24.80%	13.60%	7.00%	5.80%
Model fit statistics								
Log-likelihood	-4093.3	-3602.8	-3721.9	-3418.9	-2547.3	-2310.5	-2276.6	-2167.1
AIC	8190.5	7244.5	7481.9	6911.9	5098..6	4657	4591.1	4404.1
BIC	8204.1	7379.7	7610.3	7161.9	5112.1	4778.7	4719.6	4640.7

Discussions

This study was conducted to examine disparities and trends of ANC service utilization in urban Ethiopia over the last 20 years using Ethiopian Demographic and Health Survey data. In this study, ANC4+ utilization increased from 43.1% in 2000 to 58.7% in 2019, which means that coverage increased by 15.6 percentage points over the five survey periods. As compared to Nigeria, in this study, ANC4 utilization showed an increment while it slightly decreased in Urban Nigeria from 77.6% in 2013 to 76.1% in 2018 (30). The increment in Ethiopia may be partly explained by the implementation of the urban health extension program by the Ministry of Health and partner organizations working on maternal health in the country, through health information campaigns about the importance of antenatal care in reducing the complications related to pregnancy, but the overall level of ANC4+ utilization remain lower than the Nigerian study, this could be as result of period of the two studies.

On the other hand, the findings of this study reveal a significant decrease in the utilization of eight or more antenatal care (ANC8+) visits, from 11.4% in 2000 to 3.6% in 2019, reflecting a 7.8 percentage points reduction over five survey rounds. This trend is particularly concerning in light of global efforts to enhance maternal health. One possible explanation for this decline is the adoption of the WHO's Focused Antenatal Care (FANC) model, which was promoted during the survey period by the World Health Organization (WHO) and adopted in many low- and middle-income countries, including Ethiopia. However, in 2016, WHO revised its guidelines and introduced a new antenatal care model recommending a minimum of eight ANC contacts(5). Despite the launch of this new model, the current findings suggest that its impact had not yet materialized by the time of the 2019 survey. This may reflect a lag in policy adoption, health system readiness, or community-level awareness of the updated guidelines.

Between 2000 and 2019, notable variations in the use of ANC4 among women aged 15–49 were noted by geographic region and socioeconomic background. ANC4 utilization increased from 60.5% in 2000 to 81.8% in 2019, with an average annual change of 1.1% per year among the urban non-poor. It shows a slight change (37.8% to 48.7%) among the urban poor over the twenty years' period with an average annual change of 0.6%. This finding implies that there are urban ANC4 utilization disparities. Even if the maternal health services are provided for free in the governmental facilities of Ethiopia, women are not equally utilizing the services. Several reasons

may explain this gap. For instance, poor people spend more time on activities that generate income needed for daily living than on accessing health services(31). On the other hand, the time spent waiting in a health facility and the indirect costs related to travel to the health facility discourage poor people from seeking antenatal care. Similarly, significant associations between antenatal care and household wealth status were reported by other studies in Ethiopia(10,19). Our finding is also similar to other studies reporting that higher incomes are associated with better health-seeking practice and good health status(16). On the other hand, ANC8+ utilization showed a substantial decline among both urban non-poor and urban poor women between 2000 and 2019. Among the urban non-poor, utilization dropped from 23% to 10.5%, representing a 54.9% decrease. Among the urban poor, it declined even more sharply from 7.8% to 0.6%, marking a 92.3% reduction. These findings highlight growing disparities in antenatal carte utilization with urban population, with urban poor experiencing the steepest decline. In this study, maternal education was significantly associated with utilization of both ANC4+ and ANC8+ services. About 71.4% of women who attended at least four ANC visits and 16.4% of those who completed eight or more visits had attained a secondary or higher level of education. This finding is consistent with those of several other studies(16–19,22,24,30). This finding suggests that education can promote individual health-seeking behavior and, ultimately, increased utilization. The existing evidence indicates that educated mothers may be more likely to be informed about the benefits of antenatal care utilization.

Although many studies have emphasized the advantages of urban residence in accessing and utilizing maternal health services(16,21,23,30). the present study reveals that urban living alone does not ensure equitable utilization of antenatal care (ANC) services. The findings show a significant disparities based on wealth status. Specifically, 74.5% of urban non-poor women accessed at least four ANC visits (ANC4+), compared to only 41.6% of urban poor women. The gap is even wider for eight or more ANC visits (ANC8+), with 20.3% of urban non-poor women meeting this threshold, while only 4.4% of urban poor women did the same. These findings highlight that, despite geographic proximity to healthcare facilities, socioeconomic inequality within urban settings remains a major barrier to accessing essential maternal health services. Wealth status clearly emerges as a stronger determinant of ANC utilization than urban residence alone. This underscores the need for targeted interventions within urban areas that address the specific challenges faced by economically disadvantaged women. In this study, no difference was

observed between currently working and not working women in ANC4+ utilization. This may indicate that ANC4+ utilization is more influenced by the economic status of the women than their current working status in the urban context. This finding is consistent with another study from Nigeria(30). In contrast, a study conducted in Delta State, Nigeria, found that employed women had significantly higher odds of utilizing antenatal care services compared to their unemployed counterparts(32).

There was a marked disparity in antenatal care (ANC) utilization across subnational regions. Addis Ababa had the highest coverage, with 83.0% of women receiving ANC4+ and 31.1% receiving ANC8+, far surpassing other regions. Relatively higher utilization was also observed in Tigray (69.4% ANC4+, 9.7% ANC8+), Dire Dawa (64.2%, 14.2%), and Harari (59.1%, 15.4%), though none showed a statistically significant difference compared to Addis Ababa. Still, Addis Ababa had a 19.8 percentage-point advantage over Tigray, and a 73.5% disparity was observed between the highest and lowest performing regions. At the lower end, Somali region reported only 22.0% utilization, while Gambella recorded only 1.7% ANC8+ utilization, highlighting significant regional disparities. These patterns reflect differences in infrastructure, health service availability, and socio-cultural barriers influencing maternal health-seeking behavior, particularly in underserved regions such as the Somali, Afar, and Gambella regions. Similar intra-country disparities have been observed in Nigeria and the Democratic Republic of Congo (33,34), likely reflecting similar socio-economic challenges and regional inequalities in health service delivery. Multilevel binary logistic regression showed that the age of the pregnant women, educational status of the pregnant women, child birth order, family size, marital status, wealth status, religion and region revealed statistically significant associations with four plus antenatal care utilization. For instance, pregnant women whose age lies between 30-35 years were two times more likely to utilize) than mothers who were aged 15-19 years. This is also similar for those aged between 35-39 years. The significant association between maternal age and ANC4+ utilization in this study echoes findings from urban settings in Nigeria and Kenya, where older women are more likely to seek comprehensive antenatal care due to increased health awareness and perceived benefit of frequent ANC attendance(20,35). However, the plateauing effect among women aged 45-49 years may reflect declining perceived necessity or health system engagement in later reproductive years.

The other factor that is significantly associated with ANC4+ utilization is education; mothers who attended secondary and higher levels of education were nearly three times more likely to utilize ANC4+ than mothers with no formal education. This finding is consistent with studies from Tanzania and Uganda, where higher education attainment among women directly correlated with increased health service uptake(36,37). The higher odds of ANC4+ utilization among women with secondary or higher education suggest that education not only empowers women to make informed health choices but may also facilitate better utilization of health care and increased health literacy.

The wealth status of a woman is also significantly associated with ANC4+ utilization. Urban non-poor women were almost twice more likely to utilize AN4+ than urban poor women. The positive association between urban non-poor and ANC4+ utilization is in line with the study conducted in Ghana and South Africa, where economic empowerment translated into greater access to health services(16,38). In contrast, urban poor women face economic barriers despite geographic proximity to health facilities, underscoring the importance of addressing indirect costs like transportation and opportunity costs related to healthcare access. On the other hand, women from the urban poor often engage in various income-generating activities to cope with the high cost of living in urban areas. The need to earn a livelihood can severely limit their availability and ability to access maternal health services, including antenatal care. As a result, poor women may prioritize immediate financial needs over their health, leading to inadequate use of antenatal care. Media exposure is significantly associated with ANC4+ utilization. Women who were exposed to media at least once per week were 1.52 times more likely to utilize ANC4+ than their counterparts. This finding aligns with findings from Malawi and Rwanda, where regular media exposure has been shown to increase health knowledge and promote positive health-seeking behavior(21,39). This highlights the importance of health communication strategies in urban settings to bridge knowledge gaps and encourage ANC4+ utilization.

Other factors significantly associated with ANC4+ utilization were marital status, childbirth order, and family size. Marital status played an important role, married women were found to be 2 times more likely to utilize AN4+ compared with those who were never married. This finding is also supported by the study from Kenya(20), which suggests that marital support may enhance access and the likelihood of seeking maternal health services. Women experiencing their first birth were 1.55 times more likely to engage in ANC4+ utilization compared to those having six or more

children. This could indicate that first-time mothers may be more motivated to seek medical support during pregnancy, possibly due to a lack of prior experience(16). On the other hand, women with a smaller family size were 1.21 times more likely to utilize ANC4+ than those with six or more family size. This could indicate that large family sizes might create resource constraints or reduce the perceived importance of extensive prenatal care(38). Overall, these factors highlight the complex interplay between social and family dynamics in shaping maternal health service utilization, particularly the utilization of ANC4+.

The analysis also revealed several key determinants associated with the utilization of eight or more antenatal care (ANC8+) visits. Maternal age was positively correlated with increased ANC8+ utilization. Women aged 30–34 years and 40-44 years had nearly twice odds of ANC4+ utilization compared to the reference group of 15–19 years. This trend aligns with findings from other studies(32) indicating that older women are more likely to seek comprehensive antenatal care. On the other hand, Maternal education was a significant predictor of ANC8+ utilization. Women with higher educational attainment were more likely to attend eight or more ANC visits. This is consistent with existing literature that underscores the role of maternal education in enhancing health-seeking behaviors(20,40). In line with this wealth Status played a crucial role in determining ANC8+ utilization. Women from urban non-poor had higher odds of attending eight or more ANC visits, reflecting the influence of financial resources on access to healthcare services(38)

Women who follow Protestant, Muslim, or other religions exhibited reduced odds of utilizing ANC4+, with Muslim women showing particularly lower odds of attending ANC8+ visits. This may be linked to cultural or religious norms that prioritize same-sex healthcare providers. These preferences, combined with potential gender imbalances in the healthcare workforce, could contribute to lower utilization of ANC services within the religious communities. These are also supported by different studies(41,42).

Generally, this study highlights the multifaceted nature of ANC utilization in urban Ethiopia, influenced by individual, economic, and regional factors. Addressing these disparities requires tailored interventions that enhance health education, reduce economic barriers, and improve service delivery, particularly in underserved regions. Future research should explore context-specific strategies to enhance equitable access to maternal health services across diverse urban settings.

Strengths and limitations

The Ethiopian Demographic Health Survey is a nationally representative household survey implemented regularly and provides health information nationally, and data is analyzed using a multilevel model to identify the individual and community level factors. However, EDHS data collections were subject to several sources of bias. One of them is reliance on self-reports of ANC service utilization, with the possibility of recall bias.

Conclusions

This study showed a positive trend in the utilization of four or more antenatal care (ANC4+) visits among urban women, increasing from 43.1% in 2000 to 58.7% in 2019. However, the utilization of eight or more ANC visits (ANC8+) declined significantly, from 11.4% in 2000 to 3.6% in 2019. These trends underscore persistent disparities in ANC utilization based on wealth status, educational level, marital status, media exposure, and geographic location. To improve ANC4+ and ANC8+ utilization, particularly among urban poor women, targeted intervention, media awareness creation, and educational initiatives should be prioritized. Additionally, addressing economic barriers and fostering collaboration among stakeholders will be crucial in promoting equitable access to antenatal care services.

List of abbreviations

ANC	Antenatal Care
AOR	Adjusted Odds Ratio
EDHS	Ethiopian Demographic Health Survey
FMoH	Federal Ministry of Health
HSDP	Health Sector Development Program
SDG	Sustainable Development Goal
SPH	School of Public Health
SSA	Sub-Saharan Africa
UHC	Universal Health Coverage
VIF	Variance Inflation Factors
WHO	World Health Organization

Declarations

Ethics approval and informed consent

This study used data freely accessible from the five EDHSs. All EDHS surveys received ethical approval from the respective organization. The protocols followed the regulations of the U.S. Department of Health and Human Services for the protection of human participants. Additionally, written ethical approval for the study was obtained from the institutional review board of the College of Health Sciences of Addis Ababa University with protocol no.040/24/SPH

Consent for publication

Not applicable

Data availability

The data set analyzed in this study is publicly available from the DHS website (https://dhsprogram.com/)

Competing interest

All authors declare that they have no competing interests.

Funding

No funding received for this study

Authors' Contributions

WD contributed to the design and conception of the study and analyzed and interpreted the data and prepared the manuscript**. WM** and **DM** participated in data analysis and the interpretation and review of the manuscript. All authors read and revised the draft manuscript and approved the final version.

Acknowledgements

The authors would like to thank major DHS center for sharing the data used for the analysis for free. We would also acknowledge Addis Ababa University, school of Public Health and individuals who were directly or indirectly participated in the preparation of this manuscript.

Authors' information

Worku Dechassa, Assistant Professor of Reproductive Health, Department of Reproductive, Family and Population Health, School of Public Health, College of Health Sciences, Addis Ababa University, Ethiopia

Wubegzier Mekonnen, Associate Professor of Public health, Department of Reproductive, Family and Population Health, School of Public Health, College of Health Sciences, Addis Ababa University, Ethiopia

Desalegn Y. Melesse, Assistant Professor of public health at Institute for Global Public Health, University of Manitoba, Winnipeg, Canada

Reference

1. World Health Organization(WHO). Improving maternal and newborn health and survival and reducing stillbirth. 2023.
2. World Health Organization (WHO). Maternal mortality Fact sheet. Vol. 13, The American Journal of Surgery. 2023. p. 574–5.
3. Alkema L, Chou D, Hogan D, Zhang S, Moller A-B, Gemmill A, et al. National, regional and global levels and trend in MMR between 1990 and 2015 with scenario-based projections to 2030; a systematic analysis by the United Nations Maternal Mortality. Lancet. 2016;387(10017):462–74.
4. World health Organization. Trends in maternal mortality 2000 to 2020: estimates [Internet]. WHO, Geneva. 2021. Available from: https://www.who.int/reproductivehealth/publications/maternal-mortality-2000-2017/en/
5. World Health Organization (WHO). WHO recommendations on antenatal care for a positive pregnancy experience. 2016.
6. Kifle D, Azale T, Gelaw YA, Melsew YA. Maternal health care service seeking behaviors and associated factors among women in rural Haramaya District, Eastern Ethiopia: a triangulated community-based cross-sectional study. Reprod Health [Internet]. 2017;14(1):1–11. Available from: http://dx.doi.org/10.1186/s12978-016-0270-5
7. Barros Fernando C, Zulfiqar AB, Maneesh B, N HT, G Victora Cesar RCE and GRG. Global report on preterm birth and stillbirth (3 of 7): evidence for effectiveness of interventions. BMC Pregnancy Childbirth. 2010;10(S1):1–36.
8. Darmstadt GL, Bhutta ZA, Cousens S, Adam T, Walker N, De Bernis L. Evidence-based, cost-effective interventions: How many newborn babies can we save? Lancet. 2005;365(9463):977–88.
9. Awasthi S, Agarwal S. Determinants of Childhood Mortality and Morbidity in Urban Slums in India. Indian Pediatr. 2003;40(12):1145–61.
10. Dwomoh D, Iddi S, Afagbedzi SK, Tejedor-Garavito N, Dotse-Gborgbortsi W, Wright J, et al. Impact of Urban Slum Residence on Coverage of Maternal, Neonatal, and Child Health Service Indicators in the Greater Accra Region of Ghana: an Ecological Time-Series Analysis, 2018–2021. J Urban Heal [Internet]. 2023;(0123456789). Available from: https://doi.org/10.1007/s11524-023-00812-0
11. World Health Organization and UNHabitat. Global report on urban health: equitable, healthier cities for sustainable development. 2016; Available from: http://www.who.int/about/licensing/copy-right_form/index.html
12. FMOH. Health sector Transformation plan II 2020-2025. Vol. 25. 2024.
13. Moller AB, Petzold M, Chou D, Say L. Early antenatal care visit: a systematic analysis of regional and global levels and trends of coverage from 1990 to 2013. Lancet Glob Heal [Internet]. 2017;5(10):e977–83. Available from: http://dx.doi.org/10.1016/S2214-109X(17)30325-X
14. Tekelab T, Chojenta C, Smith R, Loxton D. The impact of antenatal care on neonatal mortality in sub-Saharan Africa: A systematic review and meta-analysis. PLoS One [Internet]. 2019;14(9):1–15. Available from: http://dx.doi.org/10.1371/journal.pone.0222566
15. Tekelab T, Chojenta C, Smith R, Loxton D. Factors affecting utilization of antenatal care in Ethiopia : A systematic review and meta- analysis. 2019;1–24.
16. Andegiorgish AK, Elhoumed M, Qi Q, Zhu Z, Zeng L. Determinants of antenatal care use in nine sub-Saharan African countries: a statistical analysis of cross-sectional data from Demographic and Health Surveys. BMJ Open. 2022;12(2):1–13.
17. Woldegiorgis MA, Hiller J, Mekonnen W, Meyer D, Bhowmik J. Determinants of antenatal care and skilled birth attendance in sub-Saharan Africa: A multilevel analysis. Health Serv Res. 2019;54(5):1110–8.

18. Tripathi V, Singh R. Regional differences in usage of antenatal care and safe delivery services in Indonesia: Findings from a nationally representative survey. BMJ Open. 2017;7(2).
19. Rosário EVN, Gomes MC, Brito M, Costa D. Determinants of maternal health care and birth outcome in the Dande Health and Demographic Surveillance System area, Angola. PLoS One. 2019;14(8):1–19.
20. Adow I, Mwanzo I, Agina O, Wanzala P, Kariuki J. Uptake of antenatal care services among women of reproductive age in Mandera County, Kenya. Afr J Health Sci [Internet]. 2020;33(1):56–69. Available from: https://indexmedicus.afro.who.int/AIM/opac_css/doc_num.php?explnum_id=73592
21. Wang Y, Etowa J, Ghose B, Tang S, Ji L, Huang R. Association between mass media use and maternal healthcare service utilisation in malawi. J Multidiscip Healthc. 2021;14:1159–67.
22. Ali SA, Dero AA, Ali SA, Ali GB. Factors Affecting the Utilization of Antenatal Care among Pregnant Women in Moba Lga of Ekiti State, Nigeria. Int J Tradit Complement Med. 2016;2(2):41–5.
23. El-Khatib Z, Odusina EK, Ghose B, Yaya S. Patterns and predictors of insufficient antenatal care utilization in Nigeria over a decade: A pooled data analysis using demographic and health surveys. Int J Environ Res Public Health. 2020;17(21):1–14.
24. Guliani H, Sepehri A, Serieux J. Determinants of prenatal care use: Evidence from 32 low-income countries across Asia, Sub-Saharan Africa and Latin America. Health Policy Plan. 2014;29(5):589–602.
25. Central Statistical Agency. Ethiopia DHS 2000. 2000;1–299. Available from: http://www.dhsprogram.com/pubs/pdf/FR118/FR118.pdf
26. Central Statistical Agency, ORC Macro. Ethiopia Demographic and Health Survey 2005. Heal San Fr [Internet]. 2005;(September):[446]. Available from: http://www.measuredhs.com/pubs/pdf/FR179/FR179[23June2011].pdf
27. Central Statistical Agency [Ethiopia], ICF International. Ethiopia Demographic and Health Survey 2011. 2011;1–452.
28. Central Statistical Agency Addis Ababa E, ICF TDP, Rockville, Maryland U. Ethiopia Demographic and Health Survey key indicators. 2016. 59 p.
29. Rutstein SO, Johnson K. The DHS Wealth Index. DHS Comp Reports No 6. 2004;(March):1–71.
30. Adewuyi EO, Auta A, Adewuyi MI, Philip AA, Olutuase V, Zhao Y, et al. Antenatal care utilisation and receipt of its components in Nigeria: Assessing disparities between rural and urban areas—A nationwide population-based study. PLoS One [Internet]. 2024;19(7):1–27. Available from: http://dx.doi.org/10.1371/journal.pone.0307316
31. Faye CM, Wehrmeister FC, Melesse DY, Kavao M, Mutua K, Maïga A, et al. Large and persistent subnational inequalities in reproductive , maternal , newborn and child health intervention coverage in sub- - Saharan Africa. 2020;1–9.
32. Rolle R Ahuru, Omon JI. Predictors of Antenatal Care Utilization in Primary Healthcare Centers in Eight Rural Communities in Delta State , Nigeria . African J Heal Econ. 2019;8(June):1–22.
33. Ononokpono DN goz., Odimegwu CO bb. Determinants of Maternal Health Care Utilization in Nigeria: a multilevel approach. Pan Afr Med J. 2014;17(Supp 1):2.
34. Okoli C, Hajizadeh M, Rahman MM, Khanam R. Geographical and socioeconomic inequalities in the utilization of maternal healthcare services in Nigeria: 2003-2017. BMC Health Serv Res. 2020;20(1).
35. Adedokun ST, Uthman OA. Women who have not utilized health Service for Delivery in Nigeria: Who are they and where do they live? BMC Pregnancy Childbirth. 2019;19(1):1–14.
36. Ngowi AF, Mkuwa S, Shirima L, Ngalesoni F, Frumence G. Determinants of Focused Antenatal Care Utilization Among Women in Simiyu Region Tanzania. SAGE Open Nurs. 2023;9.
37. Sserwanja Q, Mukunya D, Nabachenje P, Kemigisa A, Kiondo P, Wandabwa JN, et al. Continuum of care for maternal health in Uganda: A national cross-sectional study. PLoS One [Internet]. 2022;17(2 February):1–16. Available from: http://dx.doi.org/10.1371/journal.pone.0264190

38. Dahab R, Sakellariou D. Barriers to accessing maternal care in low income countries in Africa: A systematic review. Int J Environ Res Public Health. 2020;17(12):1–17.
39. Aboagye RG, Seidu AA, Ahinkorah BO, Cadri A, Frimpong JB, Hagan JE, et al. Association between frequency of mass media exposure and maternal health care service utilization among women in sub- Saharan Africa: Implications for tailored health communication and education. PLoS One [Internet]. 2022;17(9 September):1–18. Available from: http://dx.doi.org/10.1371/journal.pone.0275202
40. Tarekegn SM, Lieberman LS, Giedraitis V. Determinants of maternal health service utilization in Ethiopia: Analysis of the 2011 Ethiopian Demographic and Health Survey. BMC Pregnancy Childbirth. 2014;14(1):1–13.
41. Okedo-Alex IN, Akamike IC, Ezeanosike OB, Uneke CJ. Determinants of antenatal care utilisation in sub-Saharan Africa: A systematic review. BMJ Open. 2019;9(10).
42. Montañez-Hernández JC, Alcalde-Rabanal JE, Nigenda-López GH, Aristizábal-Hoyos GP, Dini L. Erratum: Correction to: Gender inequality in the health workforce in the midst of achieving universal health coverage in Mexico (Human resources for health (2020) 18 1 (40)). Hum Resour Health. 2020;18(1):44.

Supporting information

S1 Figure 1: Hierarchical data structure

S2 Figure 2: Model-building process for a two-level hierarchical model.

S3 Figure 3: Trends in exposure to different forms of media outlet among women in urban Ethiopia, EDHS 2000-2016

S4 Figure 4:Regional Disparities and Trends in ANC4+ and ANC8+ Antenatal Care Utilization in Urban Ethiopia, from EDHS 2000-2019.

S5 Figure 5: Trends of ANC4+ and ANC8+ service utilization by years and wealth status in urban Ethiopia from EDHS 2000 to 2019

S6 Figure 6: Trends of ANC4+ and ANC8+ service utilization by years and educational level in urban Ethiopia from EDHS 2000 to 2019

Figure1

Click here to access/download;Figure;Fig1.tif

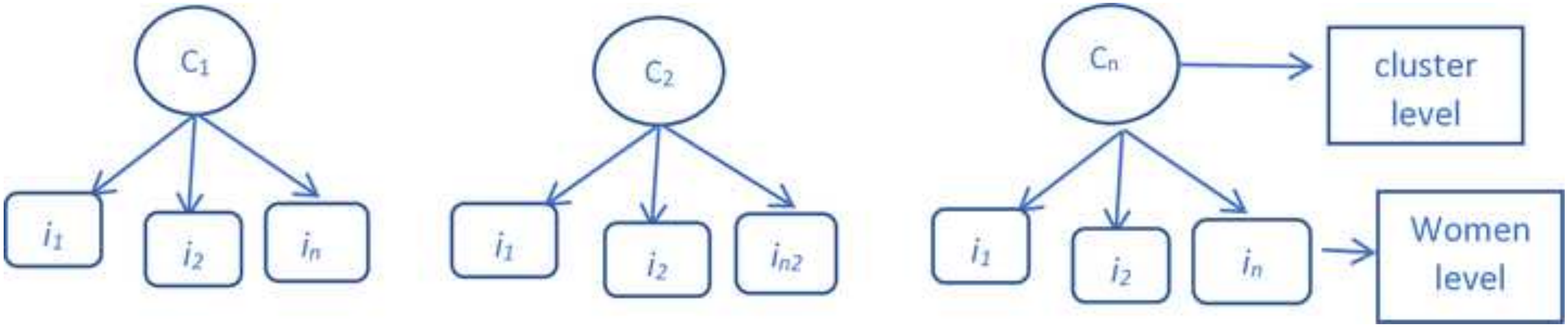

 Click here to access/download;Figure;Fig2.tif

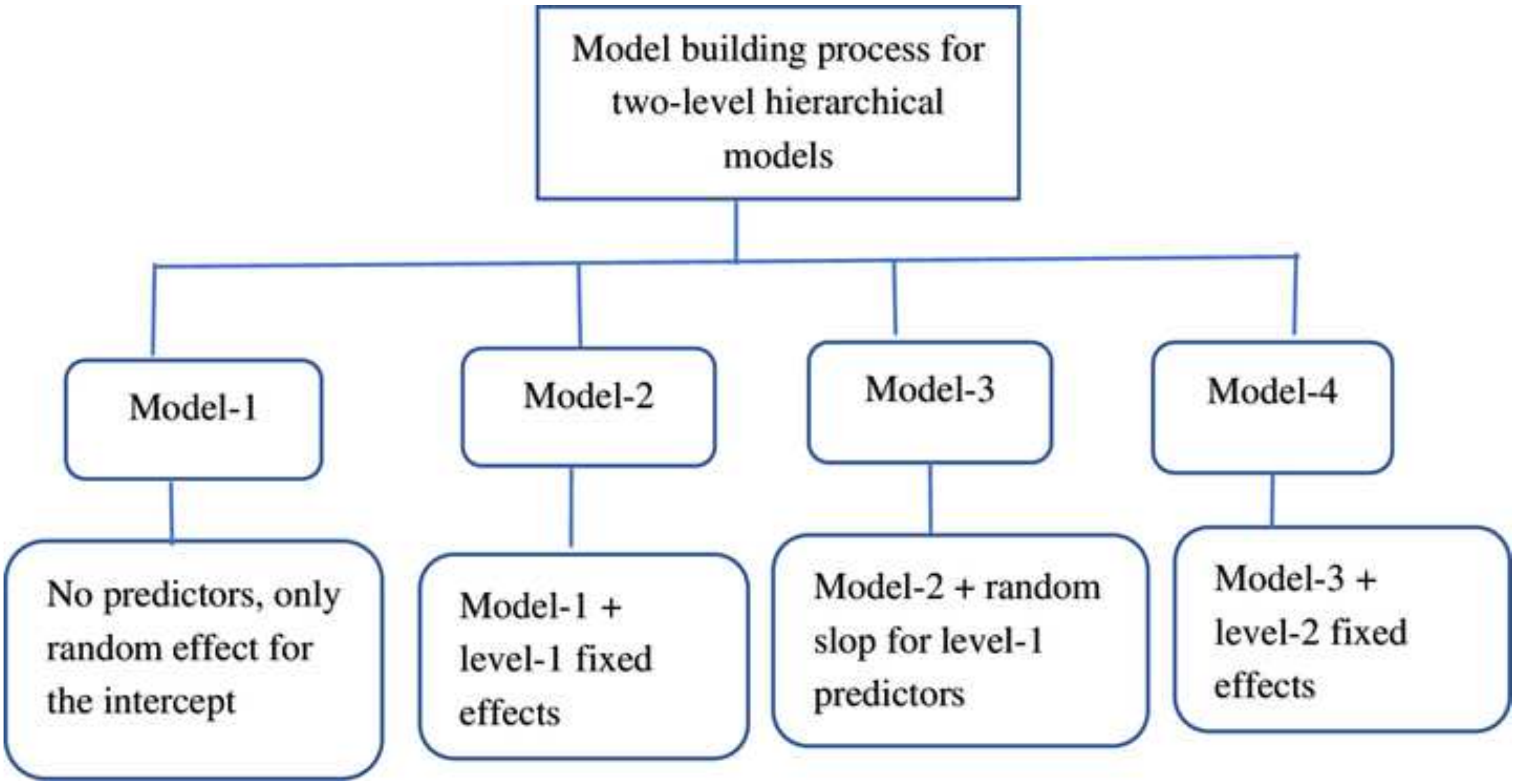

Figure3

Click here to access/download;Figure;Fig3.tif

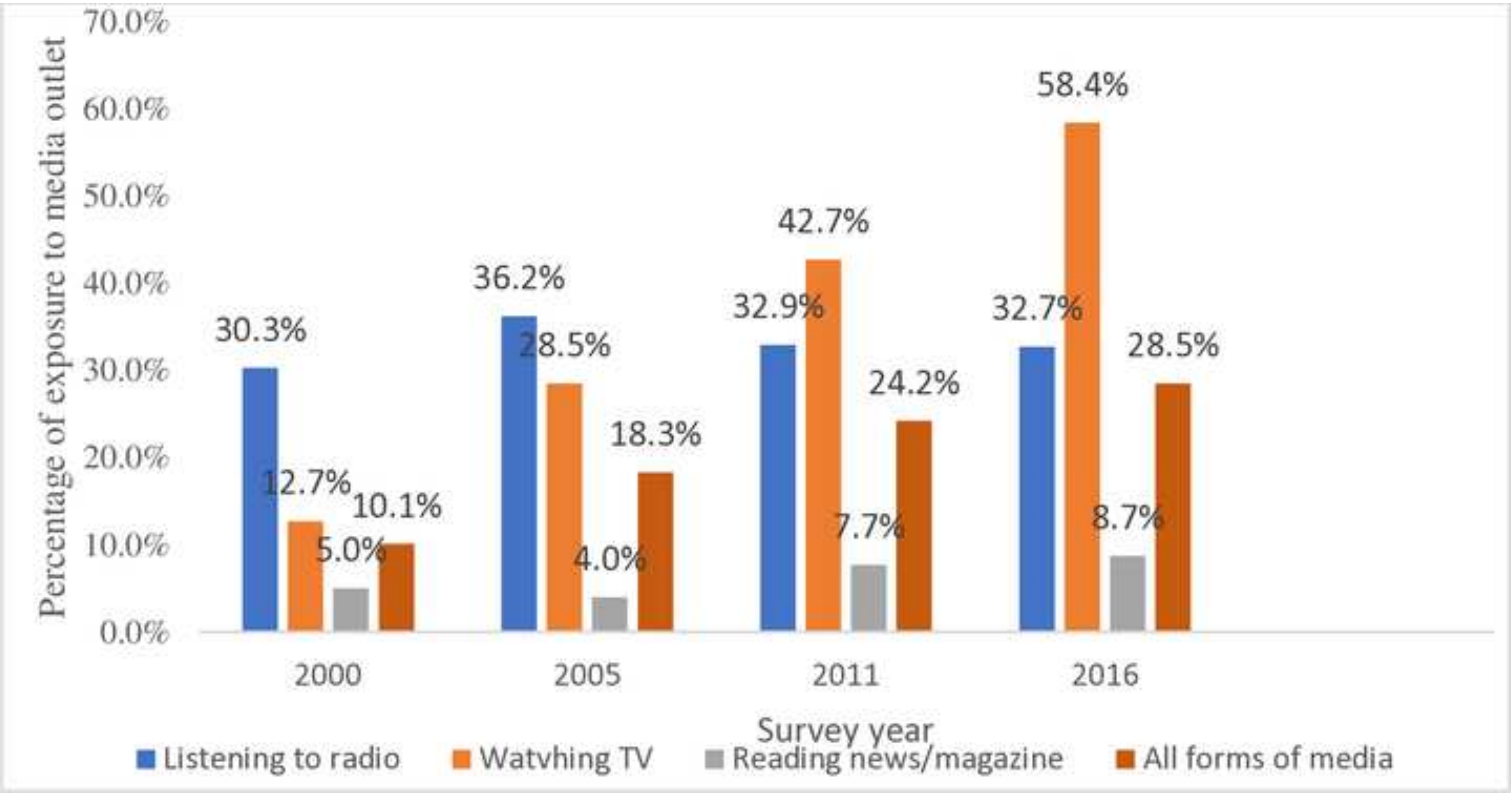

Figure4

Click here to access/download;Figure;Fig4.tif

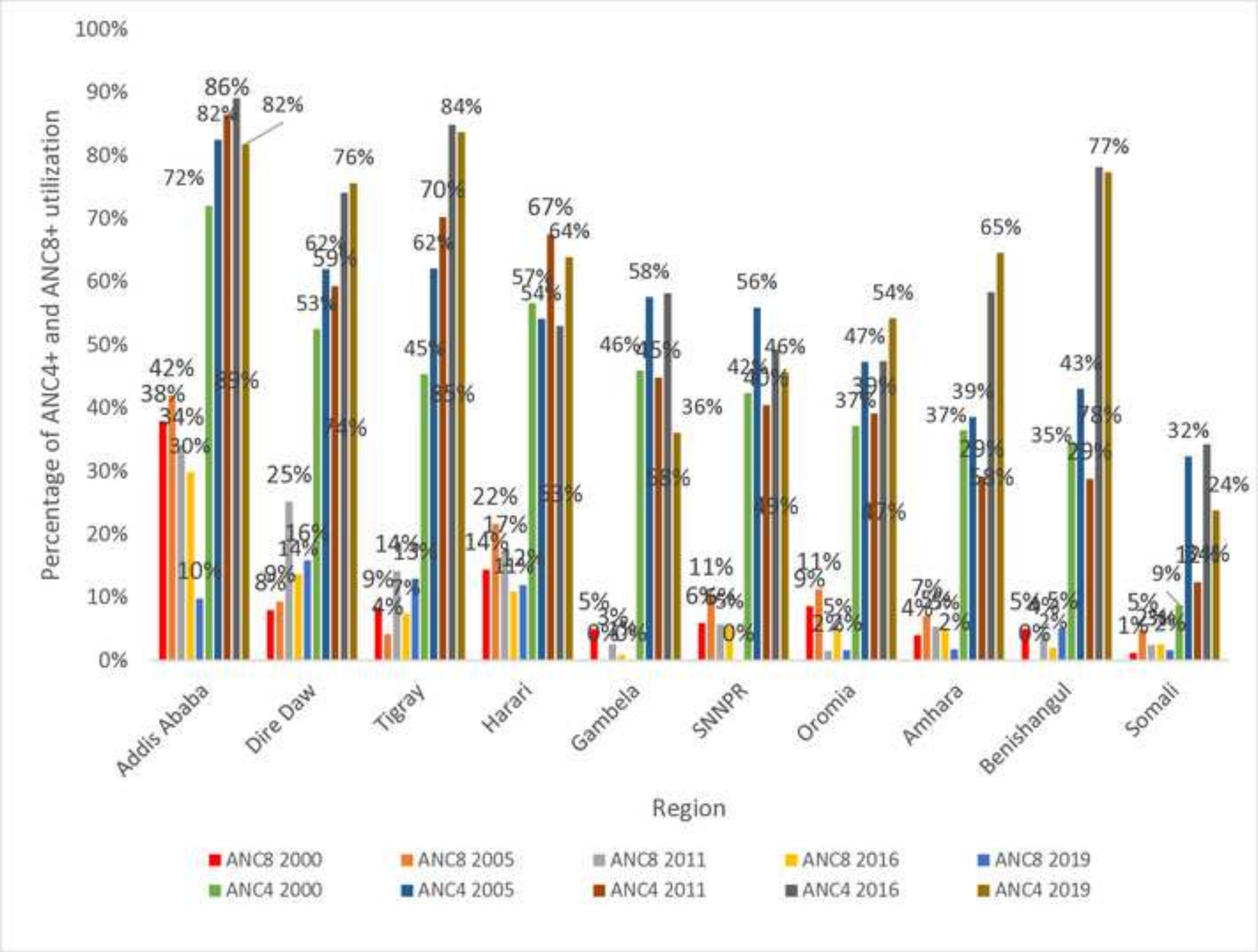

Figure5

Click here to access/download;Figure;Fig5.tif

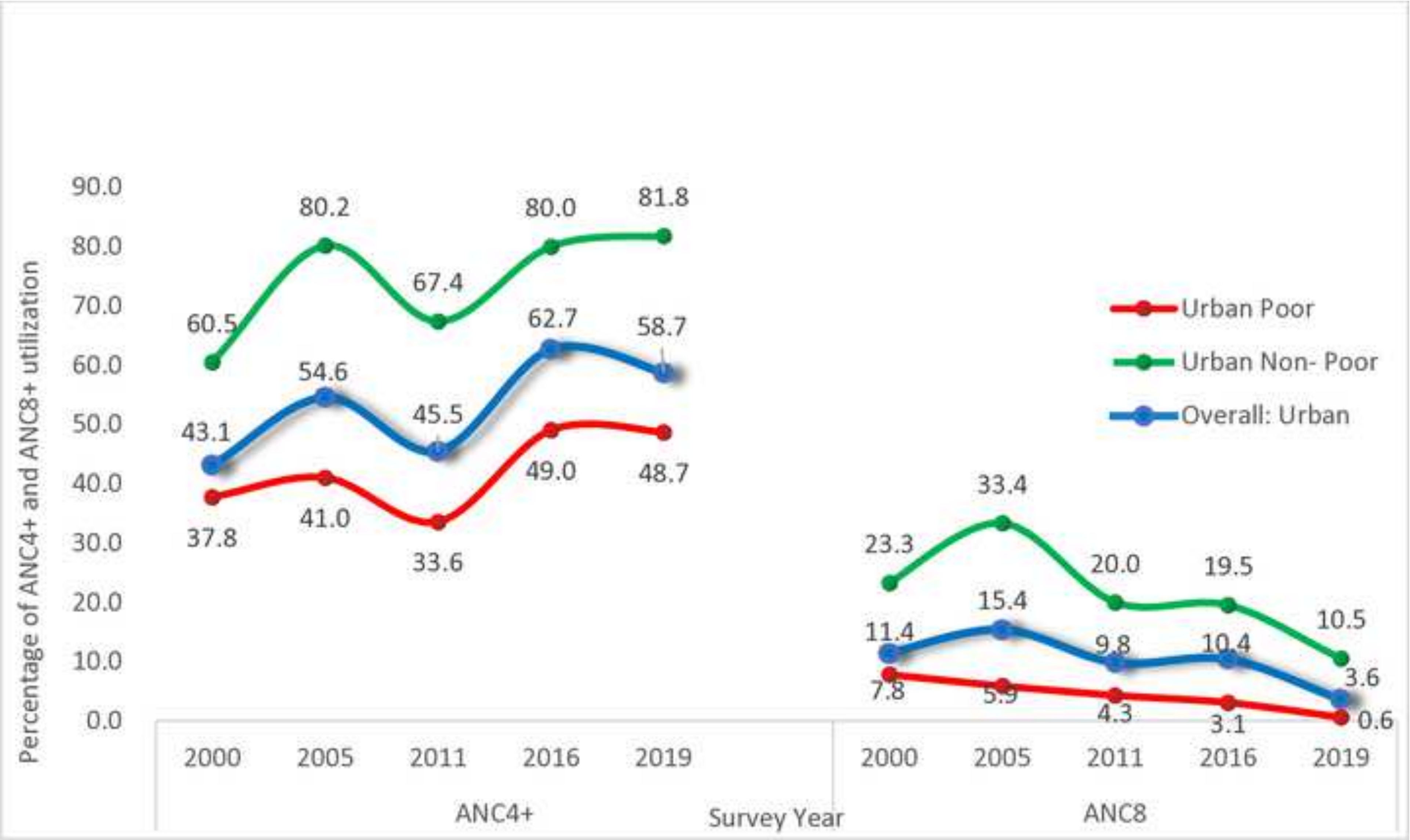

Figure6

Click here to access/download;Figure;Fig6.tif

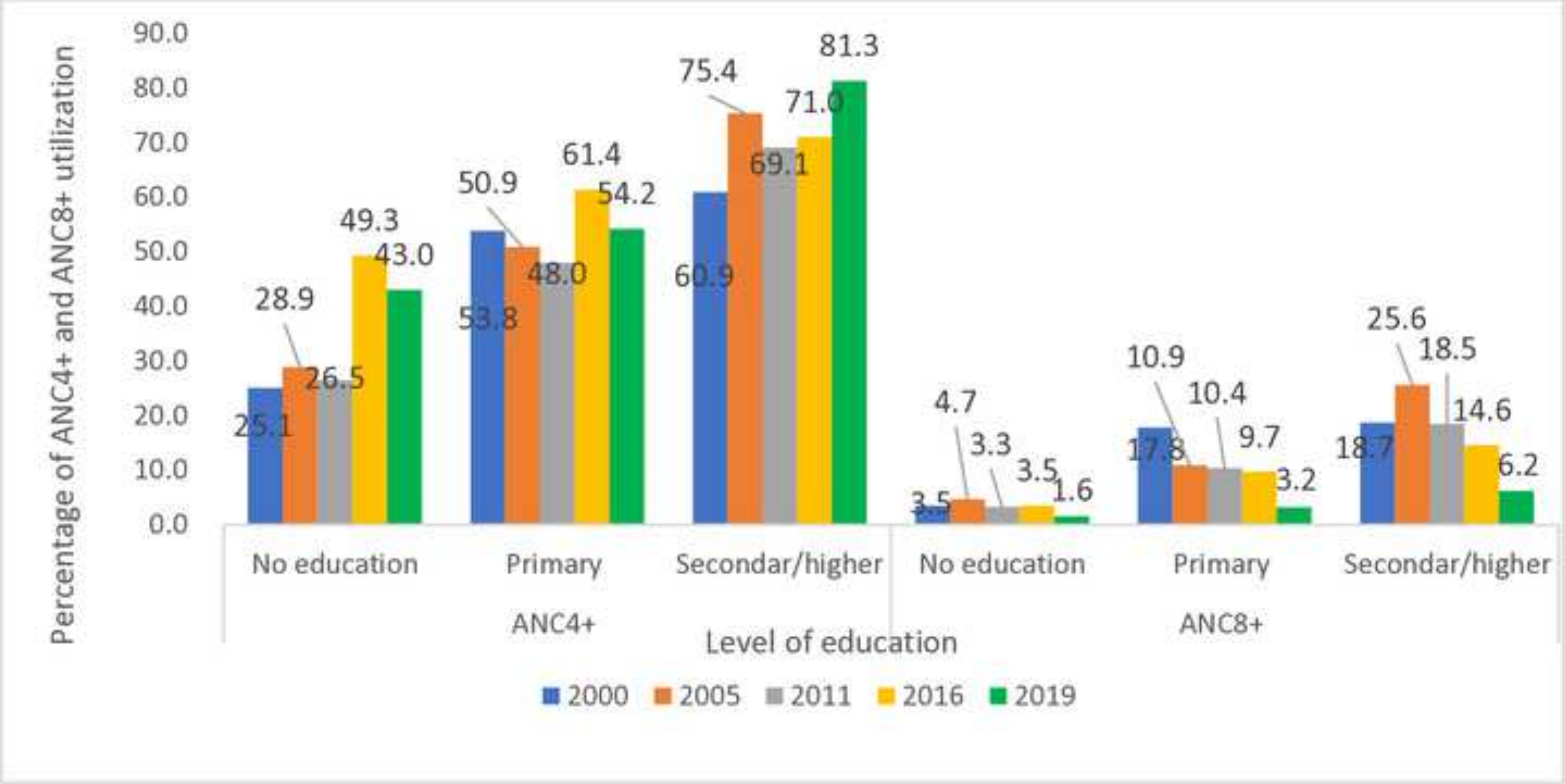

Click here to access/download
Other
IRB Approval_1.pdf
